# Current outlook on radionuclide delivery systems: from design consideration to translation into clinics

**DOI:** 10.1186/s12951-019-0524-9

**Published:** 2019-08-21

**Authors:** Oleksii O. Peltek, Albert R. Muslimov, Mikhail V. Zyuzin, Alexander S. Timin

**Affiliations:** 1 Russian Research Center of Radiology and Surgical Technologies (RRCRST) of Ministry of Public Health, Leningradskaya Street 70 Pesochny, Saint-Petersburg, 197758 Russian Federation; 2 Faculty of Physics and Engineering, ITMO University, St. Petersburg, 197101 Russia; 3 Research School of Chemical and Biomedical Engineering, National Research Tomsk Polytechnic University, Lenin Avenue 30, Tomsk, 634050 Russia

**Keywords:** Nuclear medicine, Diagnostic and therapeutic radionuclides, Drug carrier systems, Monoclonal antibodies, Nanoparticles, Microspheres, Radiopharmaceuticals, Theranostics, Multimodal imaging

## Abstract

Radiopharmaceuticals have proven to be effective agents, since they can be successfully applied for both diagnostics and therapy. Effective application of relevant radionuclides in pre-clinical and clinical studies depends on the choice of a sufficient delivery platform. Herein, we provide a comprehensive review on the most relevant aspects in radionuclide delivery using the most employed carrier systems, including, (i) monoclonal antibodies and their fragments, (ii) organic and (iii) inorganic nanoparticles, and (iv) microspheres. This review offers an extensive analysis of radionuclide delivery systems, the approaches of their modification and radiolabeling strategies with the further prospects of their implementation in multimodal imaging and disease curing. Finally, the comparative outlook on the carriers and radionuclide choice, as well as on the targeting efficiency of the developed systems is discussed.

## Introduction

Since the discovery of radioactivity by Henri Becquerel and Marie Curie, and first medical application of radium by Henri Alexandre Danlos and Eugene Bloch for the treatment of tuberculous skin lesion, the radionuclide therapy has made significant progress. Nowadays, it is hard to overestimate the importance of radionuclides application in medicine, as they are extensively used in both diagnostics and therapy [1]. The reason for such demand is explained by two main factors. First of all, the radionuclides are highly effective and universal cytotoxic agents that are capable of destroying cells with ionizing radiation, which cannot be avoided or negated by any cellular means [2]. Secondly, the use of γ and β+ emitters is crucial for diagnostics, because it allows one to take advantage of superior imaging methods utilizing the emission for precise detection of the radionuclide distribution in the whole organism. Although radionuclides possess unique diagnostic and therapeutic features, they are not able to selectively target tumor sites (with only few exceptions, e.g. ^131^I can passively accumulate at the tumor tissues, expressing sodium/iodide symporter (NIS) [3–5]), thus, issues related to the radionuclide delivery become an area of a significant interest and growth.

**Fig. 1:**
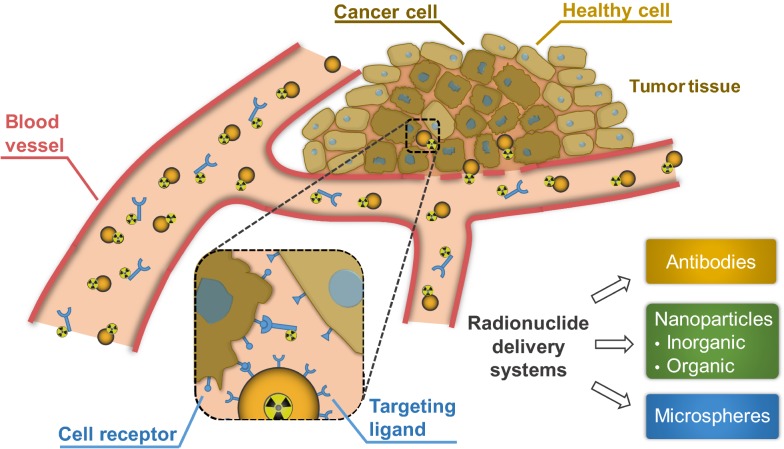
Administration of radionuclide carriers in the tumor site and their further accumulation via active targeting approach

As a matter of fact, drug delivery systems promote the concentration of the drug reaching the target site and the enhanced pharmacokinetic profiles [6]. The addressable delivery of the drug can usually occur via active or passive targeting. The passive targeting is performed via accumulation of drug carriers at a particular site due to the inherent pathophysiological, physicochemical or pharmacological factors [7–9]. While the active targeting is occurred when the drug carriers are modified with active targeting ligands, possessing a high affinity for binding to a specific cell type or tissue in the organism [10–12]. In contrast to the off-target drug delivery, the active targeting enables enhanced therapeutic efficiency and reduced side effects associated with the systemic toxicity [13]. The same principles are true when it comes to the delivery of radionuclides. The use of delivery systems, which can ensure the addressable delivery of the loaded cargo, is crucial for both therapeutic and diagnostic radionuclides. The more effective delivery of radionuclides will ensure a lowering in demand of these agents, which allow decreasing isotope dose administrated per patient to reduce a risk of exposure and costs [14–16]. Also, selective targeting of diagnostic radionuclides at the site of interest will provide increasing image quality when positron emission tomography (PET) or single-photon emission computed tomography (SPECT) is performed. The targeting delivery of radionuclides becomes the main priority since the high amounts of irradiation during the treatment in unfavorable delivery areas can result in possible side effects. Additionally, delivery systems can be modified to provide further possibilities of detection and visualization. Such augmentation can further enhance the diagnostic value of radiopharmaceuticals and is important for the delivered dose estimation when it comes to the therapeutic radionuclides. The concept of active targeting delivery of radionuclides is presented in Fig. 1.

**Fig. 2:**
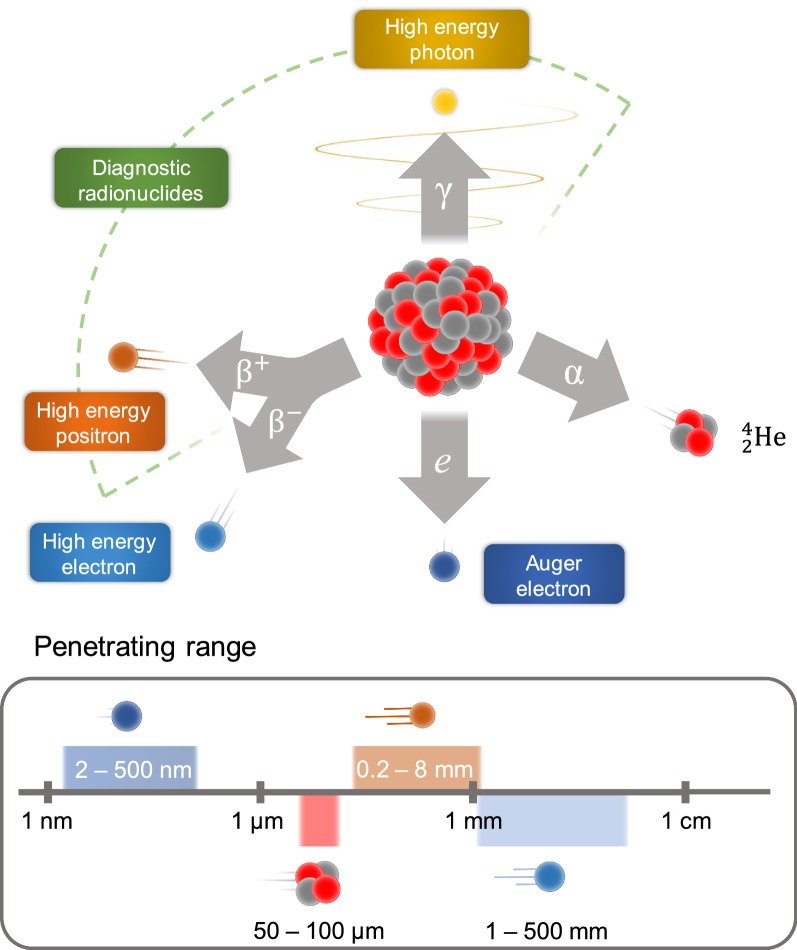
Types of radioactive decay with the demonstration of the soft tissue penetration range

The application of radionuclide is determined by its nature. Radioisotopes commonly used for the therapeutic radiopharmaceuticals emit α-, β-particles, or Auger electrons, which cause cytotoxic deoxyribonucleic acids (DNA) damage through numerous mechanisms such as reactive oxygen species, single and double stranded breaks, and inhibition of DNA repair mechanisms [17, 18]. The emissions of β-, α-, and Auger particles vary in penetrating range (Fig. 2) and linear energy transfer (LET), thus the choice of radionuclide depends on multiple factors, such as the type and size of the targeted cancer, density of the target and its heterogeneity.

The β− emitters have a low LET (0.2 keV/mm) and a relatively long penetrating range in tissue (one to several mm), as a result, they can penetrate deep in the tumor and can also partially damage the surrounding normal tissues. Common β− emitters include ^177^Lu, ^90^Y and ^131^I, which has already been used for thyroid cancer treatment. Both iodine and lutetium radioisotopes co-emit a γ photon that can be detected by SPECT [19].

The radionuclides with β+ decay emit positron and are used exclusively for diagnostical purposes and can be visualized via PET. Commonly used β+ emitters are usually short-lived (e.g.^15^O, ^13^N, ^11^C and ^18^F) and used to estimate the location of disease and quantitatively examine various biochemical and physiological processes. However, the interest in the application of the long lived β+ emitters (^89^Zr, ^64^Cu and ^52^Mn) has arisen, since they can be used to monitor the course of treatment for long period of time (2–3 weeks) [20].

The emitted α-particles can penetrate only a few cell diameters (50–100 µm in soft tissue) and have a high LET (80–100 keV/µm); therefore, they can be more effective for treatment of smaller lesions and metastases. Commonly used α-emitters include ^213^Bi, ^223^Ra, radiohalogen ^211^At, radiometal ^225^Ac [21]. One of the most frequently used in nuclear medicine radioisotopes is ^225^Ac, which consecutively undergoes four α-decays, during two of which γ ray is emitted. This isotope has been successfully applied for tumor therapy in preclinical and clinical studies [22–24]. Auger emitters (^125^I and ^99m^Tc) have high LET (4–26 keV/µm) with a very short penetration depth of 2 to 500 nm [25]. Therefore, Auger emitters like α-emitters are more suitable for minimizing damage of the normal tissues compared to β-emitters [26]. However, due to the small penetration depth of Auger electrons, the radionuclides should be delivered at the closest proximity to the cell. For this reason, the development of the radionuclide delivery systems is required.

This review is focused on known radionuclide delivery systems traditionally applied in nuclear medicine, which are categorized into four main groups: (i) antibodies and antibody fragments, (ii) organic and (iii) inorganic nanoparticles (NPs), and finally (iv) microspheres (Fig. 1). We describe the design considerations of recently developed radionuclide delivery systems, their modifications and radiolabeling approaches, as well as a current state in clinical and preclinical studies, or their potential to be implemented into the clinical practice.

### Antibodies and antibody fragments

**Fig. 3:**
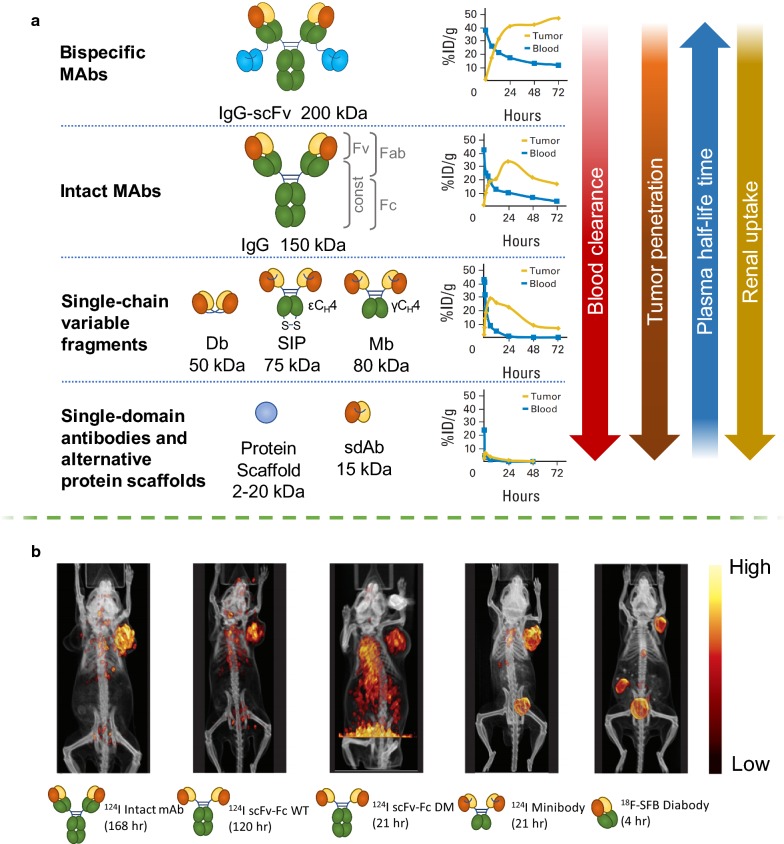
**a** Correlation between blood clearance, tumor penetration, plasma half-life and renal uptake and the previously discussed antibodies and antibody fragments of different size: bsAbs, intact MAbs, scFv, sdAbs and alternative protein scaffolds. Uptake is expressed as percentage of injected dose per gram (ID/g). **b** PET/computed tomography (CT) images of mice bearing prostate stem-cell antigen (PSCA)–expressing LAPC-9 prostate cancer xenografts. PSCA expression was visualized with intact mAbs, single-chain Fv–Fc (scFV-Fc) wild type (WT) mAbs, scFV-Fc double mutant (DM) mAbs, Minibodies and Diabody labeled with *N*-succinimidyl-4-[^18^F]-fluorobenzoate (^18^F-SFB). All microPET images were scaled individually to best show tumor targeting (this figure was reproduced from Scott et al. [29] with the required copyright permission)

Immunoglobulin type G (IgG) monoclonal antibodies (mAbs) are the most commonly used type of targeting molecules for pharmaceutical application, including radiomedicine. Their size is approximately 150 kDa, and mAbs are composed of two identical polypeptide “heavy chains” paired with two “light chains”. MAbs consist of an antigen-binding fragment (Fab), a fragment crystallizable (Fc), two disulfide bonds in the hinge region and a conserved glycosylation site at amino acid N297 of each heavy chain (Fig. 3) [27]. MAbs can specifically bind antigens without further modifications, and thus they already possess targeting properties.

The mAbs labeled with a γ- or positron-emitting radionuclide are actively used for quantitative biodistribution imaging using SPECT or PET [28]. The use of mAbs-based PET and SPECT has been implemented in cancer treatment to guide therapy selection, to estimate dosimetry for radioimmunotherapy (RIT), and to track response to therapy [29, 30]. Apart from imaging of oncological targets, immunoPET has been employed for detection and tracking of immune cells [31]. The immunoPET can also be used to monitor inflammation and immune responses [32].

Given the nature of attached radionuclide, the same antibody can be used for both diagnostic imaging and RIT, thus, providing a theranostic platform [33]. A number of works showed efficiency of RIT [34–36] in hematopoietic cancers and to a lesser extent, solid tumors [37].

Additionally, there is an approach in radiomedicine in which theranostic radionuclide pairs such as ^86^Y and ^90^Y are used [38, 39]. For instance, mAbs labeled with diagnostic radionuclides (β+ emitter ^86^Y) were administered first in order to localize the tumor site and determine the specificity of the applied mAbs. Since the ^86^Y and ^90^Y are the same element, the distribution and pharmacokinetics of mAbs labeled with these radionuclides is the same. This allows a very precise estimation of the required dosage of therapeutic radionuclide for RIT and, thus, improvement of the treatment [38, 39].

Nonetheless, mAbs possess certain disadvantages, which are common to any therapeutic radionuclide carriers. For example, a decay is a continuous process, which occurs without any regard to whether the mAbs are bonded to their antigens or not. Therefore, it is important to shorten the free circulation period of the radioconjugates as much as possible. MAbs themselves typically exhibit long circulation times in blood (which contributes to bone marrow toxicity) and a reduced diffusion into the tumoral mass, which can lead to the accumulation of radionuclides in critical organs, especially, in liver. There are some possibilities to address it such as the optimization of antibody pharmacokinetics, biodistribution, and clearance in vivo. At the present moment, antibody-based delivery optimization can be achieved using modern protein engineering, which allows to reformate intact mAbs into smaller derivatives of antibodies. These derivatives include smaller monovalent fragments such as single domain antibodies (sdAbs), diabodies, minibodies, protein scaffolds, and more complex bispecific antibodies (bsAbs). Their structures are represented in Fig. 3. The smaller antibody fragments are able to clear more rapidly from the circulation compared to the intact mAbs, resulting in better contrast images after shorter periods of time. For imaging, antibody fragments that lack the Fc region are desired due to the removal of the biological function and neonatal Fc receptor (FcRn) recycling, which allows to obtain the optimal contrast at shorter time periods. Wu et al. have shown that the reduction of the molecular weight of antibodies below ~ 60 kDa can dramatically accelerate clearance. For example, high contrast images can be obtained for small single‐domain antibodies or diabodies within the same day (4–8 h), compared to the larger minibodies, which are usually formed by the next day (24–48 h) [29]. Thus, smaller antibodies offer certain advantages regarding better pharmacokinetics. For therapeutic applications, the circulation time of the radiolabeled antibody fragments, on one hand, needs to be sufficient for the dose deposition in tumors, while limiting toxicity to normal tissues. The plasma half‐life of small fragments and scaffolds (targeting molecules of protein nature [40]) can be extended by chemical or recombinant approaches such as poly(ethylene glycol) (PEG) modification (PEGylation) or fusion to Fc domain, albumin, or albumin‐binding proteins. The influence of antibody variations created by joining antibody variable light (V_L_) and variable heavy (V_H_) domains on their plasma half-lives detected by PET is presented in Fig. 3. The tumor penetration and renal uptake is increased with the size reduction of antibodies. However, the smaller antibody fragments for RIT have a lower size than renal filtration cutoff and, therefore, they are cleared through the kidneys, which is less radioresistant compared to liver.

### Minibodies

The minibody is a bivalent dimer comprising of a single-chain variable fragments (scFv) with a human IgG constant heavy chain 3 domain (Fig. 3) with a serum half-life of 5–12 h, and due to its size (~ 80 kDa) is excreted via hepatic clearance [41]. Radiolabeled minibodies were used as diagnostic PET imaging tracers targeting carcinoembryonic antigen (CEA) [42, 43], CD8 T cells [44], CD20 B Cells, PSCA and prostate-specific membrane antigen (PSMA) [45].

Minibody, composed of the recombinant scFv of L19 antibody and the constant heavy chain 4 domain of human IgE (L19-SIP), was engineered to target extra domain B of fibronectin. ^131^I-L19-SIP demonstrated superior therapeutic efficacy compared with dimeric scFv or mAbs in teratocarcinoma tumor-bearing mice [32].

For imaging, ^124^I-L19-SIP (radretumab) was developed as an immunoPET tracer to perform patient selection in clinical trials, which later received ^131^I-L19-SIP RIT. Thus, ^124^I-L19-SIP was used to predict dosage delivered to tumors in patients with brain metastases, while ^131^I-L19-SIP was tested as RIT in combination with external beam radiation in patients with multiple brain metastases. The results showed reduction in ^18^F-fluorodeoxyglucose (FDG) uptake in common patients. Radretumab was also tested in patients with relapsed hematological cancers, where its diagnostic dose was preliminary used to determine eligibility for further treatment. In cases when tumor uptake was sufficient, three of ten patients who received radretumab showed complete response (clinical trial NCT01125085). Thus, patients with advanced lymphomas could benefit from concurrent chemotherapy.

### Diabodies

The diabody is a noncovalent scFv dimer (~ 55 kDa) in which a shortened linker prevents the V_H_ and V_L_ from self-pairing, forcing cross-pairs in trans with a second chain to form a dimer, reconstituting two binding sites (Fig. 3). Radiolabeled diabodies are widely used for high-contrast PET imaging of a variety of targets due to the improved tumor penetration compared to intact mAbs. The fast clearance of the diabodies with a plasma half-life of 2–5 h is advantageous for the same-day PET imaging of immune cell subsets, such as CD20, CD4 and CD8 T cells [46, 47], as well as solid tumors, including prostate cancer [48].

### Single-domain antibodies

Single-domain antibodies (sdAbs, 12–15 kDa) (Fig. 3) include small monomeric fragments derived from human V_H_ or V_L_ domains, linked with short peptide sequence, and has been given the commercial name Nanobody™ [49, 50]. These sdAbs have high stability, low immunogenicity and more compact structure for better targeting compared to IgG [51].

The camelid and shark domains have long complementarity-determining region 3 (CDR3), which allow for improved extension into cavities of target antigens [52]. More recent radiolabeled sdAbs studies have focused on the camelid VHH (Nanobody, registered trademark of “Ablynx NV”), and common targets include PSMA [53], CEA [54], and epidermal growth factor receptor (EGFR) [55], human epidermal growth factor receptor 2 (HER2) [56], and immune cell markers [57].

There are some nanobodies labeled with therapeutic radionuclides. For instance, the sdAbs commercially available as Nanobody 2Rs15d labeled with ^177^Lu via diethylenetriaminepentaacetic acid (DTPA) and targeting tumor biomarker HER2 was evaluated in theranostic studies. ^177^Lu-DTPA-2Rs15d was injected in mice bearing luciferase expressing ovarian cancer cell line SKOV-3 xenografts. By the day 125 tumor growth was halted, 5 of 8 mice were completely tumor-free and 3 other mice had small, but unpalpable tumors [58].

Nanobodies are also employed for diagnostics. For instance, Xavier et al. reported synthesis and preclinical validation of a novel anti-HER2 tracer, ^68^Ga-NOTA-2Rs15d, for immunoPET. The described agents were capable of highly specific accumulation in the tumor areas and reduced kidney retention compared to the previously reported Nanobodies [59]. This was achieved by the removal of histidine tag. Overall, the reported tracer proved to be safe in vivo. Further, Keyaert et al. performed clinical trials employing reported immunotracer [60]. In this work, 20 female patients with HER2-positive primary or metastatic breast carcinoma were involved. The studies revealed no adverse effects of tracer administration and the radiolabeled Nanobody was able to accumulate at HER2 overexpressing tumor sites at much higher concentrations compared to the healthy surrounding tissues. Therefore, authors continued their work in a Phase II clinical trials.

### Alternative non-antibody protein scaffolds

Alternative protein scaffolds (targeting molecule of protein nature) are easy to produce in bacteria or yeast, and are typically stable in harsh labeling conditions such as high temperature. They range from 2 to 20 kDa, which enables tissue penetration to access binding sites easier than mAbs (Fig. 3). However, the small size also results in a fast renal filtration, and certain applications may require additional engineering or other modifications to increase the plasma half-life. This can be achieved by PEGylation, Fc fusion, albumin fusion, or use of albumin binding domains [61]. A widely explored scaffold for radionuclide delivery is the Affibody (Affibody AB™), small (6.5 kDa) scaffolds composed of alpha helices originally based on the Z domain of staphylococcal protein A [62]. Affibodies rapidly penetrate tumors for early high-contrast images due to rapid extravasation and tumor penetration [63], and they are compatible with short-lived radionuclides such as ^68^Ga and ^18^F for PET, or ^111^In for SPECT. Affibody-based imaging and therapy have been developed to target EGFR [64] and HER2 [65].

RIT studies in HER2-expressing tumor models support the development of the HER2-binding Affibody Z_HER2:342_ to treat trastuzumab-resistant HER2 positive tumors. Due to the small size, the Z_HER2:342_ clears rapidly and biodistribution studies with residualizing radionuclides show high renal reabsorption. Therefore, fusion to an albumin-binding domain was used to reduce renal toxicity and increase circulation for therapeutic radionuclides [66]. For example, ^177^Lu labeled Z_HER2:342_ successfully targeted HER2 positive microxenografts as confirmed by gamma-camera imaging, and the treatment extended survival in mice with high and low HER2 expressing tumors, however, overall mortality was caused by bone marrow toxicity [67]. Another study describes biodistribution of anti-HER2 Affibody Z_HER2:395_-TCO and residualizing radiometals ^111^In and ^177^Lu [65].

### Bispecific antibodies

Antibody engineering enables novel functionality such as bispecific binding. Antibodies which contain two different antigen-binding sites in one molecule are called bispecific [68]. BsAbs can target two or more antigens resulting in improved delivery to a tumor for better imaging or therapeutic outcome. Brinkmann et al. have recently reviewed the development and clinical implementation of bsAbs [69, 70]. BsAbs can be particularly useful for radionuclide delivery as part of a pretargeting strategy. According to pretargeting approach, one arm of the bispecific targets the tumor, and the other arm recognizes a radiolabeled hapten (typically a small molecule or peptide for imaging or RIT). The bsAbs are administered first and only after sufficient time, they accumulate at the tumor and clear from the circulation. Further, the radiolabeled hapten is injected, which rapidly binds to the already pre-localized bsAbs [71]. Another effective pretargeting strategy uses a trivalent bsAb with two target-specific Fabs and an anti-histamine-succinyl-glycine Fab, which are linked to each other via disulfide bonds. Example targets for bsAbs pretargeting include CD105 and EGFR [72], CEA [73], trophoblast antigen 2 (TROP2) [74].

Example of bispecific antibodies usage for pretargeting strategy was reported in the recent work of Heskamp et al. [75]. The authors demonstrated the usage of Tri-Fab antibody TF2 consisting of anti-CEA with variable fragments against histaminesuccinyl-glycine (HSG), which allow pretargeting of radiolabeled hapten–peptide IMP-288. TF2 was used for pretargeted RIT in mice bearing CEA-positive colorectal cancer xenografts, where α-emitting hapten ^213^Bi-IMP288 was shown to be at least as effective as β-emitting hapten ^177^Lu-IMP288.

### Antibody labeling approaches

Generally, the radiolabeling of antibodies is occurred through the use of chelating agents [76–81]. The attaching chelators to antibodies is performed covalently via complementary reactive functional groups for the conjugation to each other. The *N*-hydroxysuccinimide esters (NHS), isothiocyanates (SCN) and anhydrides can be considered as the most conventional reactive electrophilic groups that can react with the ε-amino group of lysines on the antibody at alkaline conditions (pH 7.2–9). In such conditions, NHS- or SCN-containing chelators can be easily conjugated with antibodies to form strong covalent bond. After the chelator attachment is achieved, the radiolabeling is occurred via the complexation process. However, in case of conjugation reaction using NHS- or SCN chemistry, the presence of multiple amino acids in antibody structure may lead to a lack of both stoichiometric control and site-specificity [82–84]. The spontaneous chelator-antibody conjugation can decrease affinity for target receptors and provide non-optimal pharmacokinetics. Therefore, the development of more desirable chemoselective approach to bind chelators with antibodies is required and urgent.

The “click chemistry” is found to be powerful method for rapid antibody chelation that provides an appropriate site-selective conjugation of chelators with antibodies. However, this method requires the antibody pre-modification. There are many examples where the reactive “click” groups have been site-selectively introduced into antibodies [85, 86]. The site-selective conjugation methods for preparation of radiolabeled antibodies include the modification of cysteines/disulfide bonds and the glycan region of the antibody and enzyme-mediated conjugation [28].

The cysteine is widely used a single amino acid or thiol-containing small molecule for antibody modification. The most popular method of antibodies conjugation via cysteines involves so-called a Michael reaction of a thiol groups with a maleimide, resulting in formation of a succinimidyl thioether product. The maleimide groups have been widely used to incorporate the chelators via cysteine thiol groups [28]. However, maleimide-thiol modification can be unstable in vivo and, therefore, other new cysteine-reactive reagents that provide enhanced conjugate stability have been explored including employing phenyloxadiazole sulfones, dibromomaleimides, and dithiophenolmaleimides [87]. Also, monobromo maleimide has been used to generate stable antibody conjugates [88].

Considering the glycan region of the antibody, they can be chemically modified to provide site-selective attachment of chelators. One widely used example of such modification includes the generation of an aldehyde group by oxidizing the *cis*-glycol groups of terminal hexoses using sodium periodate [89–91]. Further, the generated aldehydes can react with chelators, containing amine groups to form stable imine conjugates.

The application of enzyme-mediated conjugation also provides site-specific binding of chelators to target antibodies. This approach involves the use of enzymes that recognize two complementary motifs on the antibody and the chelator functional groups [85, 92]. The enzymes catalases covalent binding of the chelator to antibody using these two complementary motifs. The β-1,4-galactosyltransferase (Y289L), transglutaminase and, sortase A are typical enzymes used in site-directed enzyme-mediated conjugation of chelators to antibodies. Such technique was applied to incorporate ^89^Zr-labeled desferrioxamine (DFO) chelator and ^64^Cu-labeled 4-(1,4,8,11-tetraazacyclotetradec-1-yl)-methyl benzole acid tetrahydrochloride (CPTA) chelator [85, 93]. Another work described the ^18^F-labeling reaction using sortase [94]. It should be noted that enzyme-mediated conjugation is occurred under mild conditions that do not denature the antibody.

Besides chelation techniques, the direct radioiodination of antibodies is a powerful method for the preparation of antibody-based radiopharmaceuticals [95]. The radioiodination can be achieved by directed radiolabeling the iodine radioisotope to tyrosines on the antibody or antibody fragment using well-established procedures that reported early starting from 1980s [96]. The good example of such radiopharmaceutical is tositumomab (^131^I-labeled anti-CD20 antibody), which received Food and Drug Administration (FDA) approval for the treatment of Non-Hodgkin’s lymphoma in 2003 [97].

In case of radioactive ^18^F, the antibody labeling is performed by directed binding to a tyrosine residue via electrophilic reaction [98]. Moreover, the incorporation of ^18^F can be achieved via conjugation of ^18^F-labeled reactive precursors, containing prosthetic groups [99].

Another interesting approach of antibody radiolabeling involves photoradiochemical methods that use light-induced antibody modification with chelators derivatized with aryl azide (ArN_3_) groups [100, 101]. Recently, Holland et al. demonstrated the photochemical conjugation of mAbs with ^68^Ga via an ArN_3_ functionalized 2-[4,7-bis(carboxymethyl)-1,4,7-triazonan-1-yl]pentanedioic acid chelate (NODAGA-PEG3-ArN_3_). The method works via a conventional two-step, preconjugation and radiolabeling pathway, and also by a one-pot, pre-radiolabeling route. As the authors reported, the speed and simplicity of this photoradiochemical route renders the method suitable for automation [101].

#### Outlooks

mAbs were historically first trial instrument to provide targeting delivery of bioactive compounds into the site of interest. Many radionuclide labeled mAbs are actively used for diagnostic and therapy of malignant neoplasms while the protein engineering opportunities allows to control plasma half-life time and biodistribution parameters of radionuclides. As a result, the change of interest from the traditional radionuclide-full antibody conjugates to the antibody-based fragments is now observed. The use of antibody derivates allow to increase the specifity, targeting ability and distribution parameters of radionuclides. Besides, employing bispecific antibodies can reduce radioisotope dosage in pretargeting strategy. Also, the antibody fragments have great potential as targeting ligands for nano- and microparticle based therapeutics with the resulting constructs demonstrating enhanced selectivity, specificity and pharmacokinetics. Combination of high specifity of antibodies based targeting ligands with high loading capacity of nano- and microparticle allows to obtain modern form of radiopharmaceuticals. Moreover, the use of NPs as a radionuclide carrier guarantee the stability of radionuclide during its delivery and save the biological properties of targeting ligands, which results in minimizing antibody-radionuclide interactions. The recent studies on radionuclide delivery using antibodies and antibody fragments were presented in Table 1.

**Table 1:** Recently studied radionuclide delivery systems based on antibodies and antibody fragments

Radionuclide	Delivery system	Labeling	Application	Comments	Refs.
^89^Z	Diabody	Chelation (DFO)	PET	Molecular imaging of CD4+ T cells throughout the body has implications for monitoring autoimmune disease and immunotherapy of cancer	[46]
^89^Z	Diabody	Chelation (DFO)	PET	PET based detection of PSMA in prostatic tumor models	[48]
^177^Lu	Nanobody	Chelation (DTPA)	RIT	^177^Lu-DTPA-2Rs15d nanobody-based targeted radionuclide therapy in mice bearing small established HER2 positive tumors led to an almost complete blockade of tumor growth	[58]
^68^Ga	Nanobody	Chelation (NOTA)	PET	The described agents were capable of highly specific accumulation in the tumor areas and reduced kidney retention comparing to the previously reported Nanobodies due to the removal of histidine tag. Overall the reported tracer proved to be safe in mouse toxicity and dosimetry studies and suggested for further clinical trials	[59]
^177^Lu	Affibody ZHER_2:342_	Chelation (maGGG and maGSG)	RIT	Successful targeting of HER2 positive microxenografts was confirmed by gamma-camera imaging, and the treatment extended survival in mice with high and low HER2 expressing tumors. However, overall mortality was caused by bone marrow toxicity	[67]
^111^In	bsAbs	Chelation (DOTA)	SPECT, RIT	In a first-in-human Phase I study, anti-CEA with anti-HSG TF2 was evaluated in patients with colorectal cancer, with ^111^In-IMP288 used in the imaging cycle and ^177^Lu-IMP288 in the following therapy cycle	[73]
^213^Bi	bsAbs	Chelation (DOTA)	RIT	The authors demonstrated the usage of Tri-Fab antibody which allowed pretargeting of radiolabeled hapten–peptide IMP-288. In vivo α-emitting hapten ^213^Bi-IMP288 was shown to be at least as effective as β-emitting hapten ^177^Lu-IMP288	[75]

### Organic NPs

**Fig. 4:**
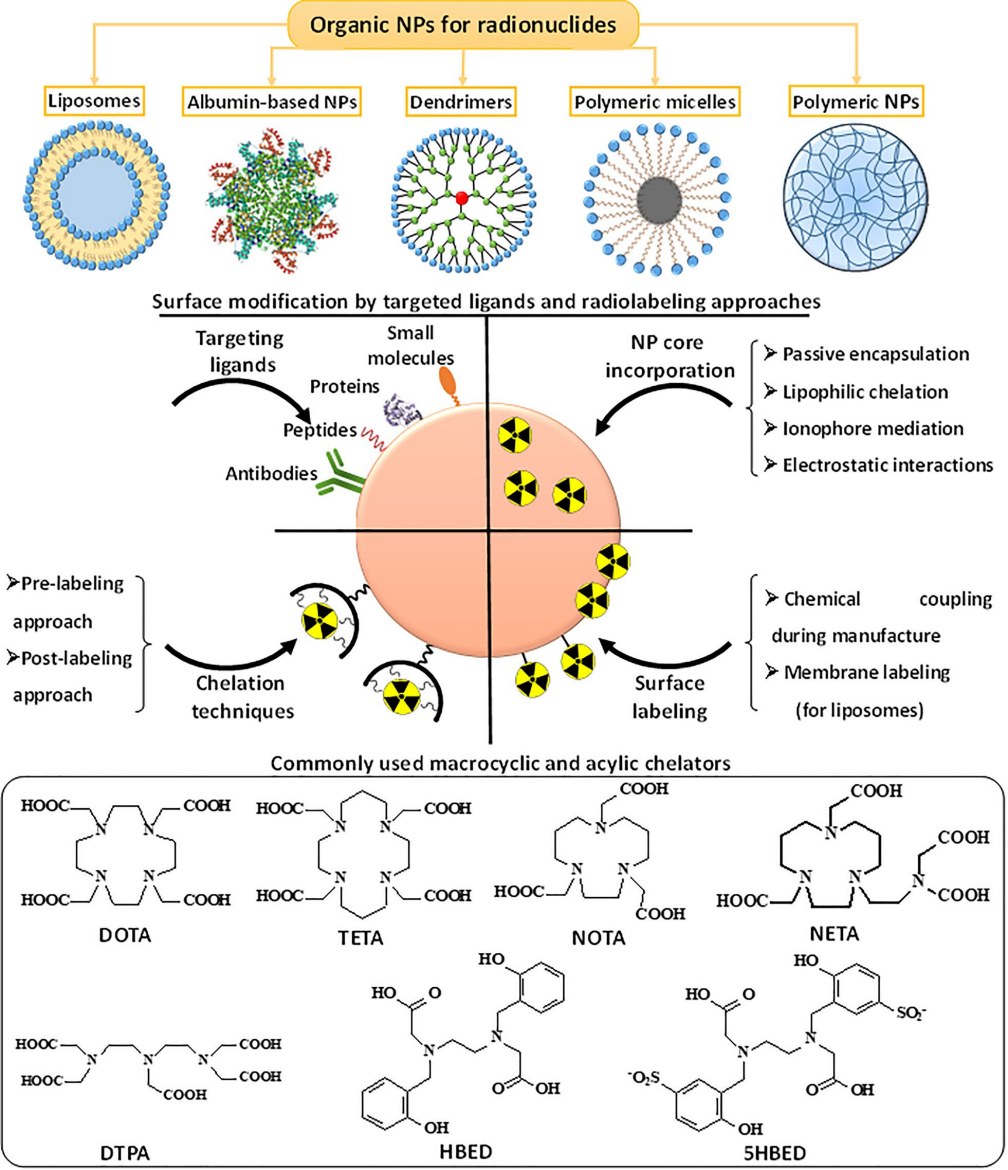
The main categories of organic NPs used for radiopharmaceutical formulations with schematic representation of their radiolabeling strategies. Below the commonly used macrocyclic and acylic chelators for radiolabeling are depicted

The organic NPs are actively used to transport various kinds of biologically active substances, including radionuclides [102]. At present, many types and variations of organic NPs were designed and fabricated. There are basically five main categories of organic NPs that have been utilized in nuclear medicine for therapy and diagnostics: (i) liposomes, (ii) albumin-based NPs, (iii) dendrimers, (iv) polymeric micelles and (v) polymeric NPs. A large number of techniques have been developed for radionuclide labeling and surface functionalization of organic NPs by targeting ligands in order to provide addressable delivery of radionuclides at the desired site, i.e., tumor area. In this section, we consider the organic nanocarriers used for radiolabeling and discuss the principles and approaches in targeting ligand modification and radionuclides incorporation with appropriate examples (Fig. 4).

### Liposomes

Liposomes are phospholipid vesicles with one or more bilipid layers that contain an aqueous phase within them. They were firstly discovered by Banghman and colleagues [103]. The sizes of these carriers can vary from the smallest (up to 100 nm) to gigantic (more than 1 micron) depending on the methods of synthesis. In the case of nanoscale liposomes, their important feature is the tendency to accumulate in the tumor tissues, providing passive drug delivery mechanism [104]. The current strategy of radionuclide delivery to the tumor sites is based on the active targeting of liposomes via their surface functionalization with specific ligands, i.e. peptides, antibodies, small molecules, proteins and so forth [105]. As an example, Anti-HER2 antibodies were successfully conjugated onto the liposome surface with conventional thiol-maleimide chemistry technique to deliver bioactive compounds into HER2-overexpressing tumor [103]. Another clinically used target is the EGFR that is constitutively activated in many solid tumors. Recently, Shargh and colleagues published the comprehensive review article describing the different conjugation techniques for preparation of antibody-targeted liposomes [103].

Cell-targeting peptides are also used to conjugate with liposomes through covalent bond by a variety of linkages (maleimide linkage bond, peptide bond, sulfanyl bond, disulfide bond and phosphatidylethanolamine-linker bond) [106]. For example, Zhang et al. reported on liposomes containing cantharidin and a tumor specific cell-penetrating peptide BR2. BR2-directed liposomes demonstrated enhanced cellular-uptake in hepatocellular carcinoma as compared to non-targeted liposomes [107]. Recently, Backer et al. developed a new method for covalent coupling of targeting proteins to liposomes via so-called “dock and lock” strategy, which exploits the natural interaction between the dimerization and docking domain that provides “safe” protein conjugation. Many small molecules (e.g. folate, affibody, carbohydrate and so forth) were used as targeting ligands for functionalization of liposome surface. However, a recent trend in functionalization of liposomes is the combination of peptides and antibodies in a single formulation for surface modification. Such dual modification provides enhanced selectivity compared to the single type of modification.

**Fig. 5:**
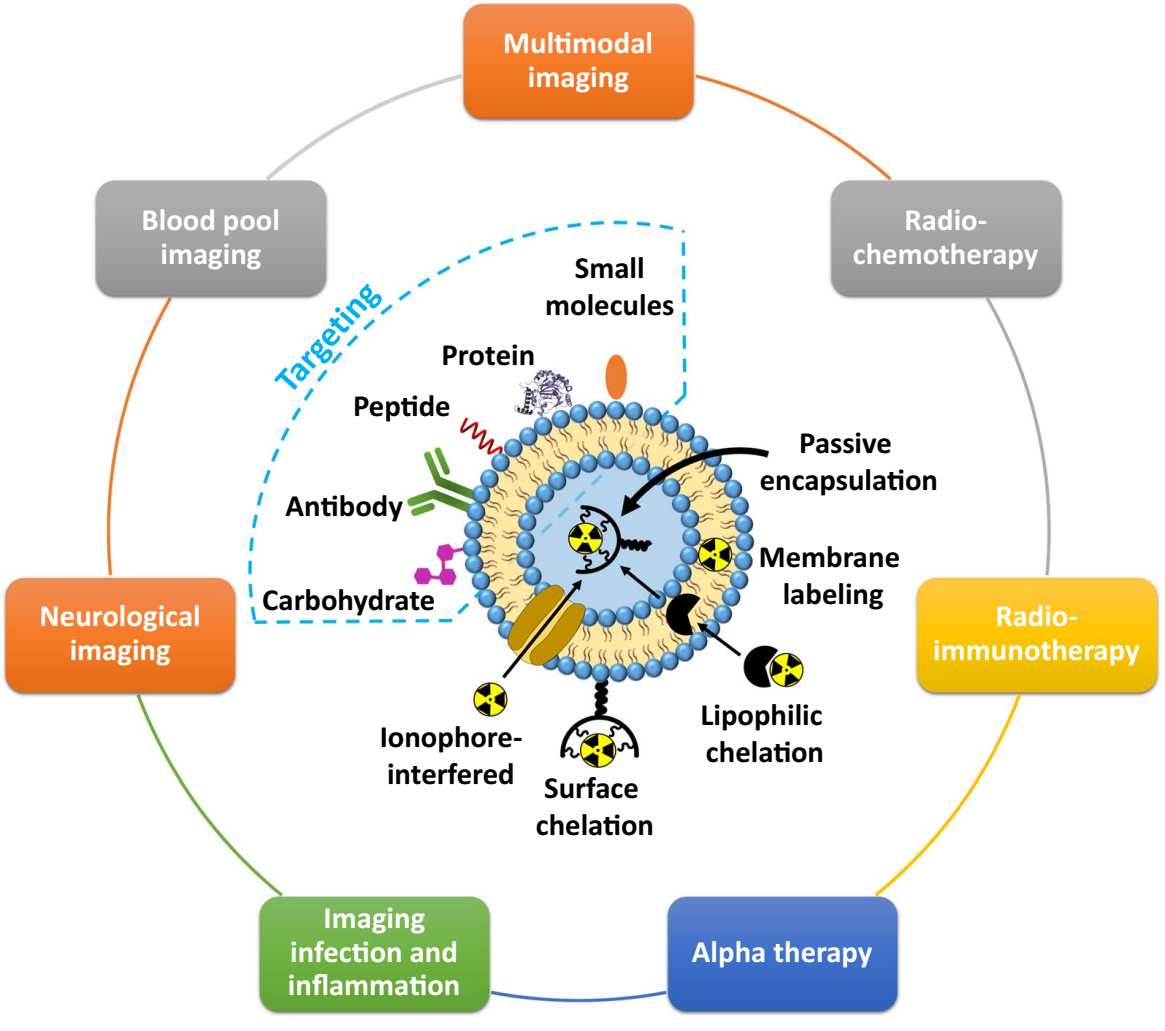
Schematic representation of various radiolabeling approaches of liposomes, targeting ligand modifications with further application in different field of nuclear medicine

In 1993 Goins with colleagues firstly investigated the labeling of liposomes with radionuclides [108]. The addition of radionuclides was performed after the liposome formation and not during the liposome synthesis. Then, the new methods of radiolabeling with liposomes have been developed. Nowadays, four general approaches can be highlighted for incorporating radionuclides into liposomes: (i) passive encapsulation, (ii) membrane labeling, (iii) surface chelation, and (iv) remote loading via lipophilic chelator or ionophore (Fig. 5) [108]. In case of passive encapsulation, the radionuclide pre-associated with chelator is included in the buffer solution, where liposomes are formed. In this method, the incorporation efficiency of radionuclides does not usually exceed 10% in case of nanoscale liposomes (< 100 nm), therefore, this method is rarely used for incorporation of highly cost therapeutic radionuclides such as ^225^Ac and ^177^Lu. According to the membrane labeling, the radionuclides can be conjugated to a lipid layer that is formulated into the liposomes via simple incubation of liposome with radionuclides. The limitation of this approach is the instability of liposome radiolabeling because of non-covalent bonding of radionuclides with liposome membrane. The use of chelating agents for radionuclide labeling can improve the stability of radionuclide biding, but not in all cases. For example, the incorporating lipid chelator conjugated onto liposome membrane does not provide an appropriate in vivo stability and the radionuclides, which are attached onto the liposome surface, interact with the components of the blood stream. This may result in the detachment of the radionuclides from the liposomes even at the labeling efficiency 90%. In general, DOTA and 1,4,7-Triazacyclononane-1,4,7-triacetic acid (NOTA) are widely used chelating agents for surface modification of liposomes. The use of lipophilic chelating agents such as *N*,*N*-bis(2-mercaptoethyl)-*N*ʹ,*N*ʹ-diethylethylenediamine (BMEDA), and hexamethylpropyleneamine oxime (HMPAO), as well as ionophores can promote penetration of radionuclides through the lipid bilayer, providing a high stability of radiolabeling [109]. Moreover, by employing lipophilic chelator or ionophore, the radionuclides can be carried through the lipid bilayer inside the aqueous phase of liposome, where the radionuclides are bound with pre-loaded metal chelator. This concept of radiolabeling has been successfully realized by Edmonds et al. Authors developed a simple and efficient radiolabeling method providing excellent radiolabeling yields, purities, and stabilities with ^89^Zr, ^52^Mn, and ^64^Cu, without the modification of the delivery system components [110].

The high flexibility of chemical structure and wide range of radiolabeling approaches allow to design the liposomes for various biomedical applications, including multimodal imaging, radiochemotherapy, RIT, α therapy, infection and inflammation imaging, neurological imaging and so forth [110]. Recently, Kleynhans et al. reported on a detailed description about application of liposomes in different fields of nuclear medicine [111].

Apart from conjugation with radionuclides, liposomes can additionally modified to provide imaging properties. As an example, Kim et al. designed trimodal liposomes for optical, nuclear, and magnetic resonance imaging (MRI) [112]. These liposomes were capable of incorporating ^124^I emitting both nuclear and optical imaging signals, and simultaneously lipophilic gadolinium complex as MRI contrast agent. Considering the theranostic approach, such liposomes could be additionally loaded with antitumor drugs (e.g. doxorubicin, vincristine and so forth). The combination of therapeutic radionuclides (e.g. ^186/188^Re) with chemotherapeutic agents (e.g. doxorubicin) in one single liposome provided radiochemotherapeutical effect [113, 114]. Interesting results were obtained for liposomes labeled with ^225^Ac, which is promising therapeutic radionuclide for targeted α-therapy [115–118]. An example for employment of radiolabeled liposomes in visualization of infection and inflammation was demonstrated by Ferreira with co-workers, who developed a long-circulating and pH sensitive liposomes containing a ^99m^Tc-labeled antibiotic (ceftizoxime) for identification of osteomyelitis foci [119]. Also the ^99m^Tc-labeled liposomes were used as blood-pool imaging agents [120]. A novel application of liposomal ^18^F is the imaging of synaptic density in the brain by targeting voltage-dependent calcium channels (N-type Ca^2+^). This tracer can image neurodegeneration and evaluate cognitive function during therapy [121].

**Fig. 6:**
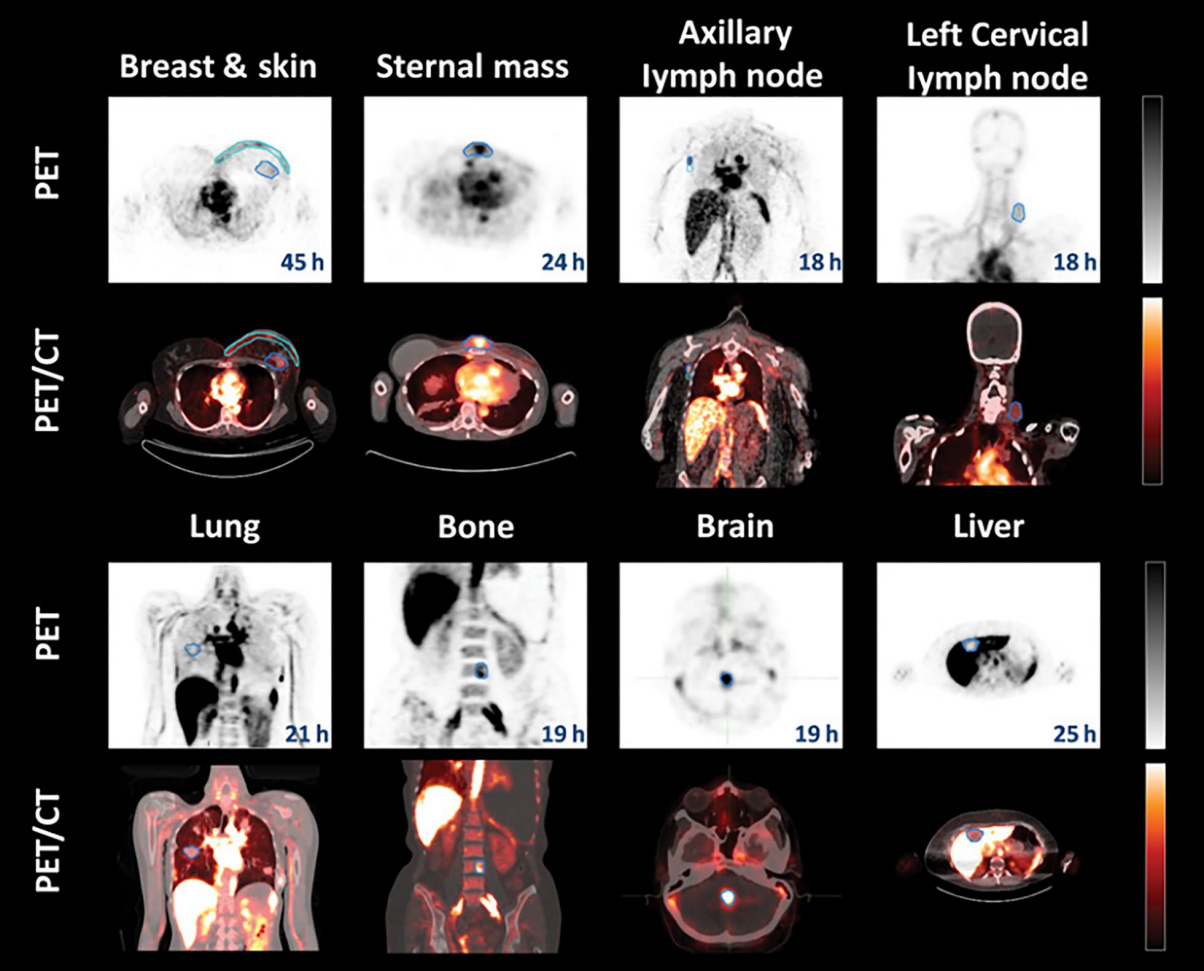
PET and PET/CT images of [^64^Cu]MM-302 in lesions at different anatomic locations. The regions of interest used to measure tumor deposition of [^64^Cu]-MM-302 are shown in blue or turquoise outlines (this figure was reproduced from Lee et al. [127] with the required copyright permission)

The liposome technologies are the most studied delivery systems in humans [122]. We refer to recently published review articles by Petersen et al. [108] and Lamichhane et al. [123], which highlighted the examples of the clinical studies focused on the application of liposomal-based radiopharmaceuticals in nuclear imaging using SPECT or PET. For example, Lopez-Berstein et al. administrated ^99m^Tc-labeled liposomes to seven cancer patients as imaging agents for tumor detection and staging [124]. In addition, there are clinical studies with ^111^In-labeled liposomes that demonstrated their safety and utility for tumor detection [125]. Later, Koukourakis et al. performed the first clinical studies of ^99m^Tc-labeled liposomes containing a therapeutic drug (doxorubicin) on seven patients with Kaposi’s sarcoma and head and neck cancer [126]. Recently, Lee et al. reported a clinical trial of MM-302, a PEGylated liposomal doxorubicin, labeled with ^64^Cu radioisotope targeted against HER2 ([^64^Cu]MM-302) [127]. The 19 patients with metastatic breast cancer for imaging study were selected for testing MM-302 radiolabeled with ^64^Cu. The results from the PET imaging (Fig. 6) demonstrated that [^64^Cu]MM-302 remained in the circulation for over 24 h, and thereafter accumulated mostly in the liver and spleen.

Another recent example of clinical use of liposomal radiopharmaceuticals was performed by the Institute of Nuclear Energy Research (Taiwan) that has already advanced a lipid theranostic agent into Phase I clinical testing (clinical trial NCT02271516). The tested liposome system was radiolabeled with ^188^Re as therapeutic and diagnostic isotope linked to BMEDA, which acts as a chelating agent. These ^188^Re-BMEDA liposomes exhibited a higher therapeutic efficacy in rodent xenografts [111].

### Albumin-based NPs

The albumin is a major protein in blood plasma and actively used in formation of albumin-based drug delivery systems because of selective accumulation into the solid tumor via so-called the enhanced permeation and retention (EPR) effect [128]. At the moment, there is already existed the first albumin based antineoplastic agent approved by the FDA, which is called Abraxane. Especially, human serum albumin (HSA) and bovine serum albumin (BSA) have driven extensive research in nuclear medicine [129]. Since the HSA molecule is more preferable than BSA for further preclinical and clinical application due to the low immunogenicity, we further review only HSA-based formulations. The HSA molecule has active chemical centers for radionuclide labeling (carboxyl, amine and thiol groups) [130]. HSA was covalently labeled with various radionuclides (^68^Ga, ^64^Cu, ^89^Zr and so forth) by employing different chelators. ^99m^Tc-labeled macroaggregated albumins are already commercially available as SPECT imaging agents such as ^99m^Tc-Pulmolite^®^ and ^99m^Tc-HSA microspheres B20^®^ for lung perfusion imaging; ^99m^Tc-Albures^®^ for liver and spleen imaging and ^99m^Tc-Nanocoll^®^ as well as ^99m^Tc-Nanotop^®^ for bone marrow and sentinel lymph node (SLN) imaging. Several techniques can be employed for effective radiolabeling of albumins. Usually, introduction of macrocyclic or acyclic chelators (DOTA, DTPA) into albumin structure is used to achieve the desired stability and suitable pharmacokinetic properties. Another promising approach to label radionuclides with HSA is the employment of low molecular weight albumin-binding molecules (HSA binders), which were further identified as reversible albumin binders. These HSA binders possess a high affinity towards HSA and can be coated with targeting ligands and radionuclides, forming HSA-binding conjugates. The concept of using reversible HSA-binders to modulate the blood circulation time and hence the pharmacokinetic profile of radiolabeled molecules was adapted and applied by other groups [128]. Besides HSA in an individual form, the radionuclide (usually ^99m^Tc) labeled HSA aggregates are widely used in clinics. For example, ^99m^Tc-labeled HSA-based NPs were prepared by radiolabeling of ^99m^TcO_4_^−^ to protein using SnCl_2_ as the reducing agent at a wide pH range of 3.8–8.0 [131]. The size of formed HSA-based aggregates depended on the amount of initial reagents and reaction time, and varied from nanometers to micrometers. The HSA-based NPs, which are generally formed via cross-linking reaction with glutarealdehyde, can be decorated with active targeting ligands and simultaneously labeled with radionuclides. The radiolabeling HSA-based NPs were also prepared using ^99m^Tc(CO)_3_^+^ precursor, possessing the high affinity binding to the histidine residues on the protein surface [132]. The more recent method for obtaining radiolabeled HSA NPs was reported by Charkavarty et al. [133]. Authors developed one-pot synthesis protocol fabrication of small sized (4–5 nm diameter), water-soluble, intrinsically radiolabeled ^64^Cu metal NPs capped within HSA. The synthesis was based on the formation of strong complex between Cu and HSA at pH ~ 12 with the following heating at ~ 55 °C that led to the formation of ^64^Cu-HSA nanocomposites. Also Tian with co-workers developed ^131^I-labeled HSA coated manganese dioxide NPs through the mixing HSA solution with manganese chloride forming HSA-MnO_2_ NPs with further labeling radionuclide ^131^I using a standard iodogen oxidation method [134].

Currently, there is a significant interest in fabrication of multifunctional HSA delivery systems, which combine several properties in one drug carrier. For example, HSA-based NPs can simultaneously contain contrast agents for MRI imaging, radionuclides for PET, antitumor drugs for combined thermotherapy and targeted ligands for active targeted delivery.

### Dendrimers

Dendrimers can be considered as well-defined monodisperse and globular nanovectors, possessing explicit architecture and composition with highly controllable size and surface properties [135]. They consist of a core and several layers with active terminal groups. The Vögtle, Newkome, and Tomalia scientific groups firstly reported on dendrimers in the late of 1970s and early 1980s independently. Nowadays, the application of dendimers in biomedicine as drug delivery platform is rapidly growing. The design principle of dendrimers, including surface/interior chemistry, size generation, shape, flexibility and composition, has been comprehensively reviewed and described in the work [136]. Various types of dendrimers including polyamidoamine (PAMAM), polypropyleneimine (PPI), poly(glycerol-co-succinic acid), poly-l-lysine (PLL), melamine, triazine, poly(glycerol), poly(2,2-bis(hydroxymethyl)-propionic acid) and PEG, as well as carbohydrate-based and citric-acid-based ones, have been developed as drug delivery platforms [137–140]. Among them, PAMAM- and PPI-based dendrimers have been some of the most widely investigated vectors that have gained tremendous attention [141]. The surface of dendrimers is rich of reactive (terminal) groups, which can be functionalized with targeting ligands. A wide selection of ligands (biotin, folic acid, amino acids, peptides, aptamers and mAbs) was successfully conjugated with dendrimers surfaces. The biodistribution of dendrimers has been investigated by administration of radiolabeled dendrimers in animals. The charge of dendrimers has a significant impact on the biodistribution and toxicity of dendrimers. As demonstrated, the cationic dendrimers possess low circulation times compared to anionic dendrimers [142]. Moreover, the cationic dendrimers were more toxic than anionic dendrimers, which can be related with membrane disruption due to interactions with negatively charged cell membranes [143].

Considering the physical half-life of radionuclides and radiolabeling strategies, dendrimers are mainly modified with chelators attached to the surface with further labeling with radionuclides (post-labeling approach) [144]. Medically relevant radionuclides were successfully applied to form stable dendrimers-radionuclide conjugates. Most of the data are associated with the preclinical studies of radiolabeled dendrimers. For example, PAMAM dendrimers functionalized with 10-[(4-carboxy-1-oxidopyridin-2-yl)methyl]-1,4,7,10-tetraazacyclododecane-1,4,7-triacetic acid (H_4_do3a-py^NO‑C^) can be mentioned. This formulation was used for radiolabeling with ^177^Lu with high radiochemical purity [145]. The pioneering work in dendrimer radiolabeling was performed by Mukhtar et al., who synthesized two water soluble dendritic porphyrins for radiolabeling with ^99m^Tc [146]. Agashe et al. also showed the potential of ^99m^Tc-labeled dendrimers using PPI dendrimers for the biodistribution investigations [147]. For solely nuclear imaging purposes, dendrimers were radiolabeled with ^76^Br to monitor angiogenesis using PET imaging [148]. It should be noted that radiolabeling with dendrimers could occur at room temperature [149]. Recently, Garrigue et al. reported on self-assembling supramolecular dendrimers bearing NOTA for complexing with ^68^Ga [150]. The ^68^Ga-labeled dendrimers showed an effective tumor targeting in case of prostate, glioblastoma, colorectal and pancreatic cancers. The radionuclide-based theranostic dendrimers labeled with radionuclides emitting both β and γ rays simultaneously (^177^Lu, ^131^I, ^188^Re) were investigated in References [151–154]. For instance, the theranostic ^131^I-labeled PAMAM dendrimers modified with PEG and chlorotoxin (CTX) as the targeting agent were employed for targeted SPECT imaging and radiotherapy of a matrix metalloproteinase-2 (MMP-2) overexpressing xenografted glioma model in vivo [152]. It should be noted that iodinated dendrimers are excellent computer tomography (CT) contrast agents, what suit for multimodal imaging (combination of SPECT with CT). The ^188^Re-labeled PLL dendrimers (ImDendrim) is into Phase I clinical testing (clinical trial NCT03255343) for treatment of colorectal cancer.

For improved diagnosis accuracy, two or more modalities of contrast imaging are combined to overcome the disadvantages of each individual technique. For this reason, radionuclide-labeled dendrimers conjugated with other imaging agents (e.g. fluorescent moieties). The development of dual SPECT/fluorescence imaging dendrimers platform is quiet useful, because radionuclide-based SPECT imaging allows increased depth penetration whereas near infrared (NIR) optical imaging provides excellent real-time spatial resolution. Urano et al. employed a PAMAM dendrimer platform that can be linked to both radionuclides and optical probes, enabling dual-modality scintigraphic and five-color NIR optical lymphatic imaging [155]. In case of PET/NIR optical imaging, Wang et al. developed PAMAM dendrimer platform simultaneously conjugated with ^64^Cu and NIR fluorescence-emitting dye Cyanine5.5 (Cy5.5) for dual-modality imaging of ovarian cancer [156].

### Polymeric micelles

The polymeric micelles are defined as organized self-assembly composed of amphiphilic macromolecules (amphiphilic, di- or tri-block copolymers) in a block-selective solvent [157]. They consist of a hydrophobic core and hydrophilic corona shell, exhibiting a wide range of sizes (usually 5–100 nm). As described in many works, the hydrophobic core serves as anchors for poorly water soluble drugs. The polymeric micelles possess the ultra-stability and can accumulate in tumor tissue through the EPR effect. A number of polymeric micelles with various structural organization as carriers for anticancer therapeutic and diagnostic agents was reported in literature [157].

Similar to other delivery systems, polymeric micelles can be functionalized with targeting ligands. Commonly used ligands are mAbs, carbohydrate moieties, aptamers, proteins, and peptides [158]. Widely used targets include the EGFR, Her2, folate receptor, and PSMA.

To incorporate radionuclide in micelle structure, the chelation techniques can be employed. In 1995, Trubetskoy and Torchilin firstly incorporated amphichilic chelating agents such as DTPA-phosphatidylethanolamine (DTPA-PE) and DTPA-stearylamine (DTPA-SA) into PEG-modified micelles and used these micelles for γ-scintigraphy of the lymphatic system after subcutaneous administration in rabbits. Nowadays, with the development of micelle fabrication, the different chelators for the radionuclide labeling can be easily conjugated with the end-groups of micelle polymeric shell. Mainly, the radiolabeling of polymeric micelles is occurred on the hydrophilic shell, while hydrophobic drugs are encapsulated in the core of the micelles. In this case, post-labeling approach is used, when chelators (DTPA or DOTA) are conjugated to the hydrophilic corona shell and further bind the radionuclides. Also, radionuclides can be encapsulated in the hydrophobic core of the micelles using a lipophilic radionuclide-ligand complex during the micelle formation. The micelles are usually functionalized with diagnostic radionuclides such as ^18^F [159, 160], ^111^In [161–164] and ^64^Cu [165–167] for further evaluation of in vivo biodistribution and targeting efficiency. There are only a few works, where micelles were labeled with therapeutic radionuclides such as ^90^Y [168], ^131^I [169]. The micelles loaded with γ emitters (^99m^Tc and ^111^In) have extensively been investigated for in vivo biodistribution studies using SPECT imaging [162, 170]. Another more relevant example was reported by Miura, who developed DOTA-functionalized polymeric micelles for labeling with ^111^In [171]. Later, Jensen with co-workers developed triblock PEG‐pHEMA‐PCMA micelle modified with macrocyclic chelator 2,2′‐(1,4,8,11‐tetraazabicyclo[6.6.2]hexadecane‐4,11‐diyl)diacetic acid (CB‐TE2A) using pre-labeling approach for complexation with radioisotope ^64^Cu. The conjugation of CB‐TE2A chelator was performed to the primary alcohols of the pHEMA block through 4‐dimethylaminopyridine–catalyzed 1‐ethyl‐3‐(3‐dimethylaminopropyl)carbodiimide coupling in dimethylformamide (DMF) [172].

In clinical trials polymeric micelles received authorization for evaluation mainly as anticancer drug carriers [173]. Almost all of them can be prepared by self-assembly of amphiphilic block copolymers containing hydrophilic PEG blocks and hydrophobic polyester, poly(aspartic acid), or poly(glutamic acid) blocks. The drug molecules used in these systems are mostly classical cancerostatics (paclitaxel, camptothecin and its analogue doxorubicin, or platinum complexes). Paclitaxel loaded polymeric micelles (Genexol PM) have reached the most advanced testing stages and received approval for the use in Europe and Korea for the treatment of breast and small cell lung cancer. Ulbrich et al. gave a clear description on clinical studies of polymeric micelles as anticancer drug carriers used in cancer therapy [173]. As mentioned above, the hydrophobic core of micelles is preferable for incorporating of hydrophobic drugs while hydrophilic corona shell suits for radiolabeling. From this point of view, these so-called theranostic micelles are generally considered as multifunctional platform that combines drugs and imaging agents for PET or SPECT.

### Polymeric-based NPs

Polymeric-based NPs can be defined as solid polymer particles or aggregates (micro- and nanosized ranges), where the bioactive compounds are encapsulated into the polymer matrix or coated with the surface of polymer NPs. There is no real classification of polymeric NPs and each literature review uses its own grouping. In this section, we did not include polymeric micelles, which have already been described above. The polymeric NPs are currently applying for different biomedical purposes, including drug and gene delivery, tissue engineering, bioimaging and so forth [174]. They are able to form highly stable complexes with radionuclides as well as permit a rapid labeling of targeting ligands. The techniques of incorporating radionuclides into polymer structure were adapted from the strategies for radiolabeling of proteins. In general, pre-labeling and post-labeling approaches are used for incorporating radionuclides with chelating agents. As for the pre-labeling method, the radionuclides are conjugated with chelating agent before their attachment to the polymer structure. In case of post-labeling approach, the polymeric NPs can be decorated with chelators via conjugation reaction or polymerization of monomers before their radiolabeling [173]. The DOTA and DTPA are most frequently used chelators that demonstrated high stability complexation with ^68^Ga, ^64^Cu and ^111^In. The attachment of chelating agents to the polymer structure is usually achieved via functional groups in chelators that can react with free amines (active esters, isothiocyanates), sulfhydryl- (maleimides, iodoacetamides), carboxylate- (amines, alcohols) or alkynyl- (azide) groups of the polymer to form stable polymer-chelator conjugates [80]. The classical organic iodination procedure is applied for polymer radiolabeling. The direct electrophilic iodination of tyrosine residues using so-called the Bolton–Hunter reagent (i.e., radiolabeled *N*-succinimidyl-3-(4-hydroxyphenyl) propionate) can be considered as the mostly varied radiolabeling method in case of polymer-based structures. Another smart and straightforward method for radiolabeling of polymeric NPs involves the formation of polyvinyl phenol particles surrounded by PEG [175]. The polyvinyl phenol is able to form a stable core, where ^125^I radionuclides can be introduced via electrophilic aromatic substitution.

Polylactic acid (PLA), polyglycolic acid (PGA) or their copolymer poly(lactic-co-glycolic acid) (PLGA) belong to the most advanced biodegradable polymers used in the preparation of polymer-based nanocarriers. Moreover, PLGA and PGA are already approved by FDA for clinical use in macroformulations. The surface of polymeric NPs is frequently coated with hydrophilic polymers (e.g. PEO) to prevent the adsorption of plasma proteins. In case of nuclear imaging, radionuclides such as ^11^C, ^18^F, ^64^Cu, ^76^Br, ^99m^Tc, ^111^In, and ^90^Y have been used with a wide range of synthetic polymers to formulate nanosized carriers [159, 176, 177]. For example, the PLGA NPs, labeled with ^99m^Tc, are successfully used in diagnostics for imaging of lungs [178] and sentinel lymph nodes [177]. ^111^In-labeled galactosylated PLGA NPs have been developed as trackable carriers for the liver specific delivery of drugs [179]. Sirianni et al. developed radiolabeled PLGA NPs with biotinylated ^18^F prosthetic groups for their tracking imaging using PET [180]. As for PLA, Banerjee et al. fabricated PSMA-targeted PLA-based NPs radiolabeled with ^111^In for SPECT imaging of PSMA-expressing tissues [181].

Natural polymers such as chitosan and dextran are also actively used for delivery of diagnostic radionuclides. Various formulations based on radiolabeled chitosan were reported. The ^99m^Tc/^131^I-labeled water-soluble chitosan derivatives were used as SPECT/PET imaging agents [182]. Akhlaghi et al. performed the radiolabeling of chitosan derivatives with ^66^Ga using chelator DTPA. The use of DTPA for complexation with ^66^Ga allowed to achieve the highest efficiency to prevent radionuclide leakage [183]. Also, Lee et al. reported a facile method to label PEOylated-chitosan NPs with ^64^Cu, using azide-functionalized chitosan and DOTA derivatives with appending dibenzyl cyclooctyne moieties. This formulation allowed fast labeling (~ 30 min) with high radiolabeling yield (98%) and showed a tumor uptake in adenocarcinomic human alveolar basal epithelial cells (A549) tumor-bearing mice. The nanosized glycol chitosan NPs were labeled with ^64^Cu via copper-free click reaction for study of in vivo biodistibution. The dextran is also effectively used for targeted delivery of radionuclides. Moreover, dextran-based carriers was proved to be effective in clinical studies (Phase I and II), and a clinical Phase III study with oral cavity squamous cell carcinoma patients confirmed the detection and accurate prediction of the pathologic nodal status with 0% false negative results [184].

#### Outlooks

At present moment, most of the described organic based NPs are used to optimize radionuclide delivery: increase circulation time, avoid non-specific activity and provide tumor accumulation. The advances in surface chemistry enable different functionalization of organic NPs with targeting ligands. Several issues should be taken into account before implementation of organic NPs into clinics. First, the appropriate and convenient approach of radiolabeling should be provided to guarantee sufficient radiochemical stability. Second, radiolabeling should not influence the particle size, shape, surface charge and composition in order to provide optimal pharmacokinetics and biodistribution. Besides, the individual characteristics of radionuclide carriers should be additionally considered: biocompatibility, biodegradability, utilization of NPs components and their cytotoxicity. The recent studies on radionuclide delivery using organic NPs were presented in Table 2.

**Table 2:** Recently studied radionuclide delivery systems based on organic NPs

Radionuclide	Delivery system	Labeling	Application	Comments	Refs.
^124^I	Liposomes	Hexadecyl4-iodo benzoate	PET, MRI, optical imaging	Kim et al. have prepared a trimodal liposome for optical, nuclear, and magnetic resonance imaging with fast clearance from reticuloendothelial systems, which enables vivid tumor imaging with minimum background	[112]
^186^Re	Liposomes	Chelation (BMEDA)	Combination of chemo- and radiotherapy	^186^Re-Doxil liposomes were used in combined therapy for the treatment of solid tumors	[114]
^64^Cu	Liposomes	Chelation (4-DEAP-ATSC)	PET	Novel ^64^Cu-MM-302 were used to quantify EPR effect in 19 metastatic breast cancer patients in order to evaluate the effectiveness of further treatment with nanoparticles	[127]
^64^Cu	HSA-based NPs	Neutron activation route	PET	Chakravarty R. et al. developed a one-pot synthesis of small sized (4–5 nm diameter), water soluble, intrinsically radiolabeled ^64^Cu metal nanoparticles capped within human serum albumin (HSA) scaffold (^64^Cu-HSA nanocomposite). Studies in melanoma tumor bearing mice showed rapid accumulation of the radiotracer in tumor with high tumor-to-background contrast	[133]
^131^I	HSA-based NPs	Iodogen oxidation method	RIT, SPECT	HSA-bound manganese dioxide nanoparticles (^131^I-HSA-MnO_2_) are developed as a RIT platform that is responsive to the tumor microenvironment. The acidic TME can trigger degradation of MnO_2_ and thus decomposition of nanoparticles into individual ^131^I-HSA with sub-10 nm sizes and greatly improves intratumoral diffusion	[134]
^111^In	Dendrimers	Chelation (SCN-Bz-DTPA)	SPECT, optical imaging	Kobayashi et al. synthesized nanoprobes with multimodal and multicolor potential, which employed a polyamidoamine dendrimer platform linked to both radionuclides and optical probes, permitting dual-modality scintigraphic and five-color near-infrared optical lymphatic imaging using a multiple-excitation spectrally resolved fluorescence imaging technique	[155]
^125^I	Polymeric-based NPs	Iodination via aromatic electrophilic substitution	PET, SPECT	Tang et al. produced polymeric NPs using Flash NanoPrecipitation and radiolabeled them with ^125^I at high radiochemical yields (> 90%). The nanocarriers demonstrated extended circulation half-lives and gradual RES clearance	[175]
^64^Cu	Polymeric micelles	Chelation (CB‐TE2A)	PET	Jensen et al. investigated the novel triblock PEG‐pHEMA‐PCMA micelle modified with macrocyclic chelator and difference of in vivo biodistribution of cross-linked an non cross-linked micelles using PET	[172]

### Inorganic NPs

**Fig. 7:**
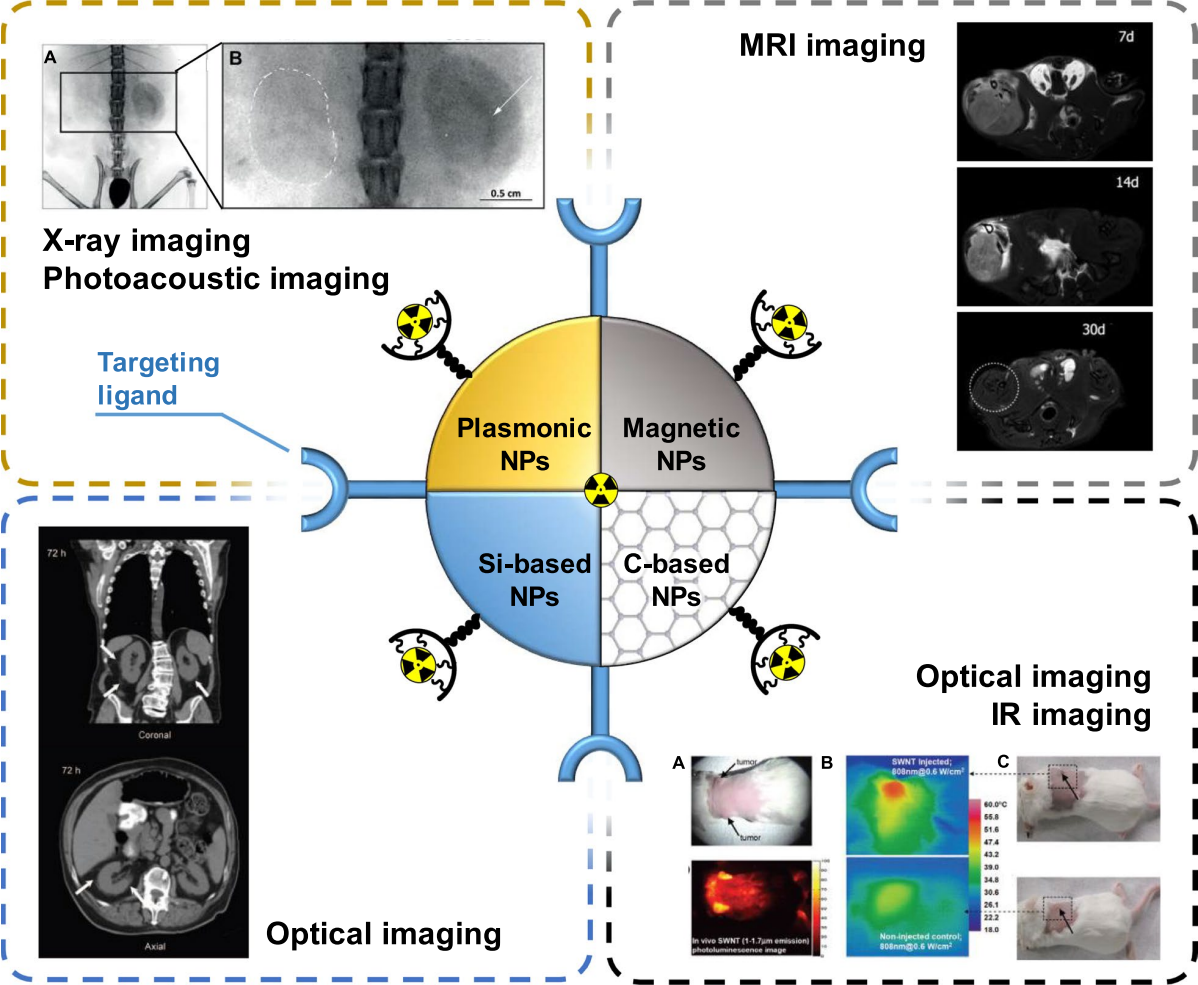
Schematic illustration of multimodal imaging of radiolabeled inorganic NPs: plasmonic NPs for X-ray visualization, magnetic NPs for MRI visualization, C-based for optical imaging. Si-based NPs requires additional functionalization by contrast agents to be visualized (this figure is adopted from Yu et al. [189], Wang et al. [190], Liu et al. [191], Phillips et al. [192], Hoffman et al. [193] with required copyright permission)

Another type of radionuclide delivery system is NPs of inorganic nature. Such NPs can consist of different inorganic materials with the sizes ranging from the nanometers to micrometers [185]. The composition and structure of the NPs core determine their unique properties. For example, the use of gold NPs (Au NPs) as a core makes it possible to perform X-ray and photoacoustic imaging using such delivery systems. Whereas the utilization of an iron core allows NPs to be used for MRI imaging (Fig. 7) [186]. Despite the unique physicochemical properties of NPs and their diagnostic potential, the use of such carriers for the delivery of radionuclides in clinical practice is rather limited. It is associated with undesired toxicity, low targeting efficiency, short circulation time in the bloodstream and so forth [187]. In this regard, only a few inorganic NPs-based delivery systems reached the stage of clinical research [188].

### Plasmonic and magnetic NPs (Au, Fe_3_O_4_)

Au NPs are widely used in biology and medicine as drug delivery carries due to their unique optical properties [194]. Indeed, pronounced localized surface plasmon resonance allows their visualization possibility in vitro and in vivo using photoacoustic [195] and X-ray imaging [196]. Optical properties of Au NPs strongly depend on their physicochemical composition. The modern chemical synthetic techniques allow fabrication of monodispersed Au NPs with well-defined size and shape [197]. Recently, Au NPs were also employed as core-particles for radionuclides in radiotherapy and radiodiagnostics.

Similar to the organic NPs, in order to achieve targeting properties of Au NPs-based radionuclide delivery systems, different moieties such as peptides, antibodies, aptamers, nucleic acids are employed. These multifunctional platforms allow targeted delivery of radionuclides with visualization of radionuclides biodistribution by PET/CT or SPECT. In the recent study, 45 nm Au NPs were integrated with radioactive ^125^I. Navigation of Au NPs-based radionuclide delivery platform to the tumor site was provided by conjugation of Au NPs surface with cyclic Arg-Gly-Asp peptide. In vivo investigation of the developed delivery system revealed acute apoptosis and tumor growth suppression of NCI-H446 tumor-bearing mice after 2 days post treatment [198]. In another in vitro comparative study authors employed 30 nm Au NPs conjugated with mAbs (panitumumab), which target breast cancer (BC) cells overexpressing EGFR. ^177^Lu was used as radioactive compound in Au-based delivery system. The obtained data showed that the EGFR overexpressing cells demonstrated lower viability than BC cells with lower EGFR expression [199]. McLaughlin et al. used so-called α generator based on Au NPs to target biologically relevant receptors. In particular, female BALB/c mouse was used as a model for the targeted delivery of radionuclides. In this case, multiple α radiations were emitted by the ^225^Ac daughters while targeting the receptors in vivo. Such radioactive generators can potentially enhance the efficiency of tumor therapy [200]. Au NPs can appear not only as a core element for the radionuclide delivery platform, but as radioactive part of the system as well. Katti et al. demonstrated a novel approach to treat prostate cancer with ^198^Au NPs, which were modified with glucoside molecules mangiferin. In vivo experiments on severe combined immunodeficient (SCID) mice revealed over 85% reduction of bearing prostate tumor volume as compared to the control groups [201].

**Fig. 8:**
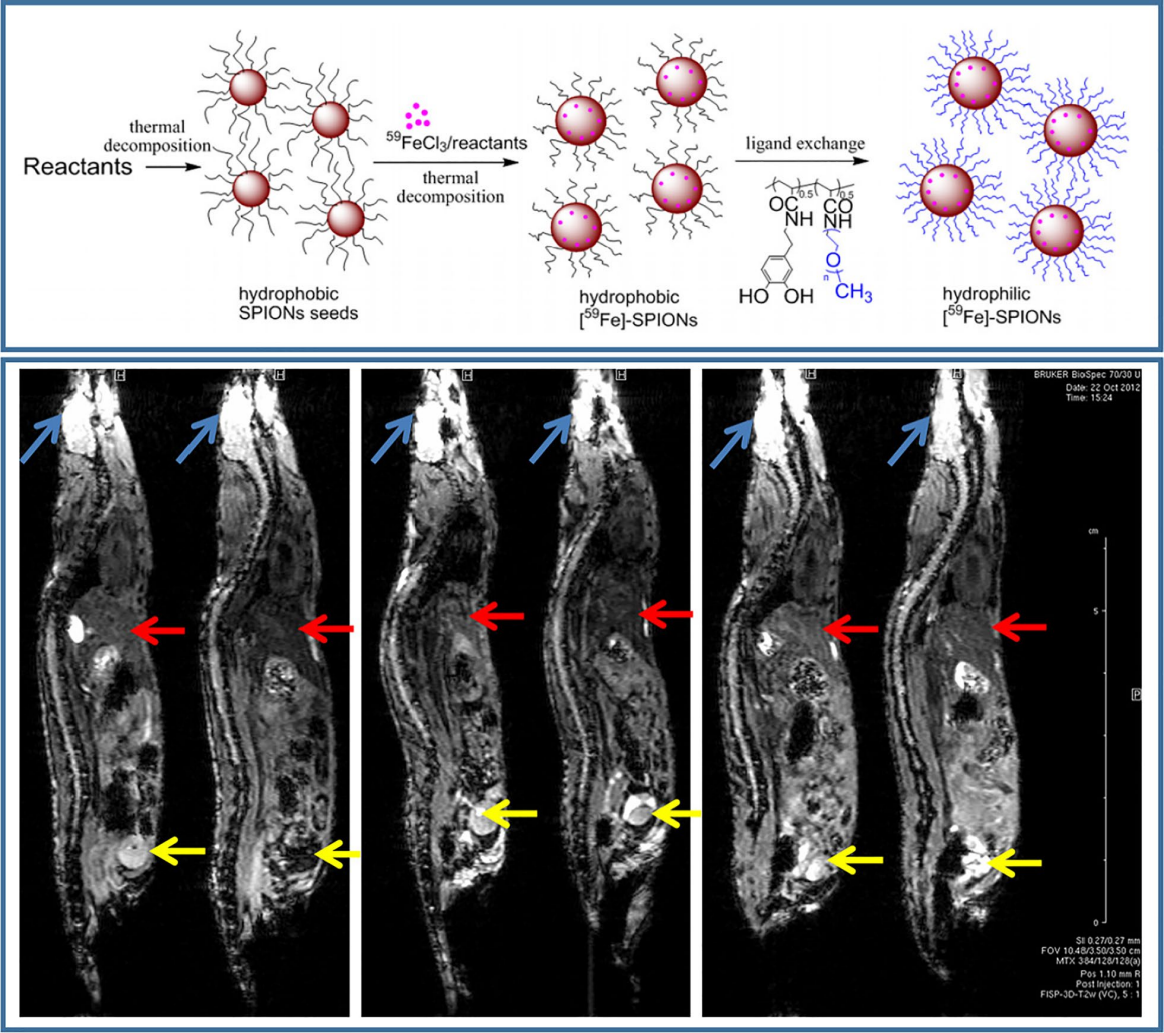
Schematic illustration of SPION-based NPs fabrication (top), sagittal MR images of mouse animal model (bottom). The blue arrows indicate the brain, the red arrows indicate the liver and the yellow arrows indicate the bladder (this figure is reproduced from Hoffman et al. [193] with required copyright permission)

Superparamagnetic iron oxide NPs (SPIONs) are also widely used as a core element for the delivery of radionuclides. SPIONs are biocompatible and clinically approved NPs [202], which enable real-time visualization of drug biodistribution with MRI. Similar to Au NPs, SPIONs can be synthesized of various shape and size that affect their MRI contrast [203], as well as interaction with cells and tissues [204]. Such different behavior was demonstrated by Radovic et al. in vivo on example of ^90^Y labeled differently sized and coated SPIONs. The biodistribution of magnetic NPs was probed on Wistar rats. The data revealed that uncoated SPIONs end up in liver in a lower rate compared to PEGylated NPs [205]. Targeting features of Fe_3_O_4_-based radionuclide delivery systems can be achieved either by surface functionalization or by applying magnet in the close proximity to cells. The latter possibility was discussed in the recent work, where magnetic NPs were modified with ^165^Ho and cytostatic drug, and navigated into tumor area using external magnet. In vitro and in vivo results suggested that the external magnetic field navigation was achieved, since higher amounts of ^165^Ho were observed in tumor than in liver, which led to the inhibition of tumor growth [206]. In another study, radioisotope ^125^I was integrated into Fe_3_O_4_-Ag heterodimer to achieve dual-modality imaging with MRI and CT. The obtained delivery platform showed high radiolabeling efficiency as well as reduced T2-MRI signal intensity [207]. Recent work demonstrated radiolabeling of Fe_3_O_4_ NPs of 20 nm size with sodium pertechnetate (^99m^Tc- pertechnetate) and the developed system was probed on male Wistar rats. The obtained results showed that this type of NPs could be introduced in the laboratory animals via intravenous injection and biodistribution could be controlled by external magnetic field [208]. Isotope ^59^Fe can be also used as radioactive element incorporated into the crystal lattice of Fe_3_O_4_ NPs. An advantage of this approach was the fact that radioactive ^59^Fe did not alter MRI contrast properties of Fe_3_O_4_ NPs. Therefore, the developed carriers enabled bimodal visualization (MRI/CT) as demonstrated in vivo (Fig. 8) [193].

### Silica-based NPs

**Fig. 9:**
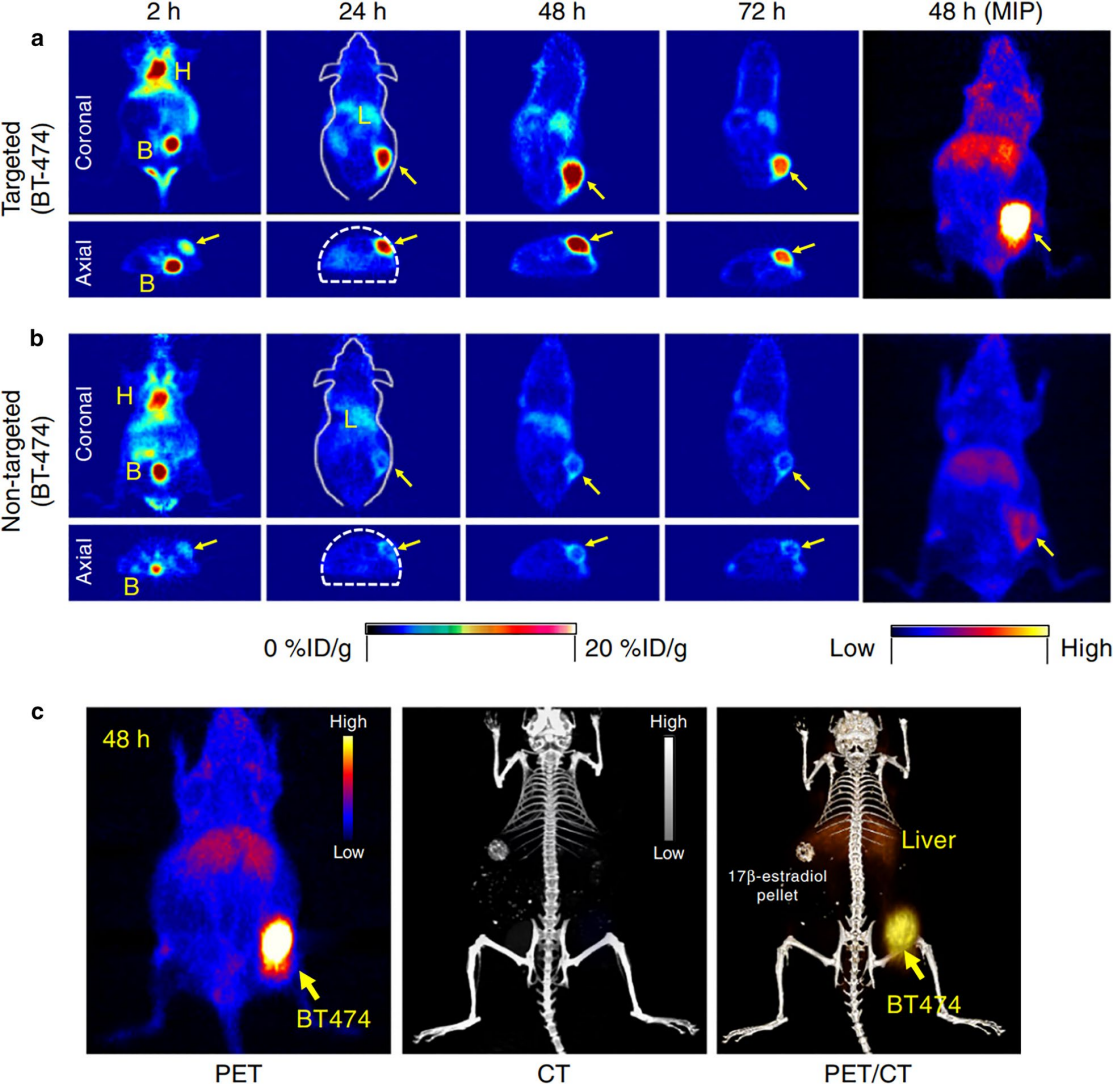
In vivo HER2-targeted PET imaging in xenograft breast cancer models. Serial coronal and axial tomographic PET images acquired at 2, 24, 48, and 72 h post i.v. injection of radiolabeled particle immunoconjugates in groups of tumor-bearing mice as follows: **a** Targeted group: ^89^ZrDFO-scFv-PEG-Cy5-C’ dots in BT-474 mice. **b** Non-targeted group: ^89^Zr-DFO-Ctr/scFv-PEG-Cy5-C’ dots in BT-474 mice. For each group, maximum intensity projection (MIP) images were also acquired at 48 h p.i. H: heart, B: bladder, L: liver. **c** Representative MIP PET, CT, and PET/CT fusion images of 89Zr-DFO-scFv-PEG-Cy5-C’ dots in a BT-474 tumor-bearing mouse. BT474 tumors are marked with yellow arrows (this figure was reproduced from Chen et al. [212] with the required copyright permission)

Silica NPs are already widely used as delivery carriers of various bioactive compounds due to the tailored physicochemical properties [209]. One of the great advantages of silica NPs is its porosity and, thus, high surface area, what makes Si NPs good candidates for employment as drug carriers. Silica NPs are usually prepared using chemical synthesis with a good possibility of size, shape and pore-size variation [210]. It is worth noting that some of silica-based materials are approved by Food and Drug Administration and generally recognized as safe [211]. Therefore, unlike plasmonic NPs, ultrasmall ^124^I modified silica-based NPs (‘C dots’) were already employed in clinical studies in 2014 on humans. Phillips et al. [192] developed radionuclide Si-based carriers, which possessed dual imaging properties: PET/SPECT and optical (due to the additional labeling with fluorescent markers). The obtained results on metastatic melanoma patients revealed good in vivo particle stability and their distinct, reproducible pharmacokinetics without any adverse effects. The development of Si-based NPs ‘C dots’ was continued in the work by Chen et al. [212]. They were able to optimize the synthesis of carriers in water and fabricated a novel PEGylated ultrasmall silica particles labeled with Cy5 dye. Si-based NPs were further conjugated with chelators and anti-HER2 scFv fragments. This allowed to achieve more efficient tumor-targeting with bulk renal clearance, which surpasses already existing characteristics of scFv molecules and scFv-conjugated NPs larger than 10 nm. The experiments were conducted in vivo on BT-474 tumor-bearing mice and revealed a favorable pharmacokinetics and clearance with decreased hepatic and reticuloendothelial system uptake (Fig. 9).

Prior first clinical trials of Si-based radionuclide-labeled carriers, series of pre-clinical studies were conducted. For example, mesoporous silica NPs of 400 nm size were used to transport stable isotope ^166^Ho in mice with orthotopic non-small cell lung cancer A549-luciferase expressing tumor. The administration of the delivery systems were intravenous and biodistribution was monitored using luciferase bioluminescence [213]. Pascual et al. improved targeting properties of developed Si-based systems by functionalization of NPs with Mucin 1 aptamer that is specific to breast cancer. Pronounced targeted delivery of ^99m^Tc into tumor of MDA-MB-231 tumor-bearing Balb/c mice was shown and monitored with SPECT and bioluminescence options [214]. In another study silica NPs were modified with anti-HER2 and labeled with ^99m^Tc. Antibody conjugation allowed to target breast carcinoma cells. Radionuclides delivery efficiency was probed in vivo on tumor xenograft models in mice. Biodistribution of the developed radioactive formulation was monitored with optical fluorescence microscopy due to the additional labeling of Si-based carriers with indocyanine green (ICG) [215].

It is worth mentioning that mesoporous silica NPs can be simultaneously loaded with various cargoes. For example, in the recent study, mesoporous silica NPs were loaded with ^89^Zr and photosensitizer Chlorin e6, which can be activated by Cherenkov radiation from ^89^Zr. Therefore, the developed radionuclide delivery systems overcame the limitations of conventional photodynamic therapy, which requires light source to activate photosensitizer [216].

### Carbon-based NPs

Carbon-based nanomaterials, which include fullerenes, carbon nanotubes (CNTs), carbon dots (CDs), nanodiamonds, graphene and its derivatives, are of significant interest nowadays due to unique optical, thermal and mechanical properties [217]. These materials can be used as drug delivery carriers, which can be simultaneously visualized by fluorescence microscopy [218]. However, there are some concerns about toxicity and biocompatibility of carbon-based materials. For example, due to the high hydrophobicity and non-biodegradability of CNTs, they possess reduced biocompatibility, what can limit their applications in biology and medicine [219]. It has significant importance in the field of nuclear medicine due to the reduced clearance of the radionuclides, what can increase the toxic effect of fabricated radioformulation. In order to overcome above mentioned drawbacks, the surface of CNTs can be functionalized with biocompatible polymers to improve their solubility and biocompatibility, thus, reduce their toxicity [220]. Therefore, recently single-walled CNTs were successfully employed to deliver radionuclides into cells. In this study, the surface of CNTs was simultaneously modified with tumor-specific mAbs, chelating agent (for radiolabeling with ^111^In) and fluorescence markers. The biodistribution ^111^In labeled CNTs was possible to monitor with fluorescence microscopy. Such multifunctional CNT-based delivery platform demonstrated effective targeting features with simultaneous visualization due to fluorescent moieties [221]. In another study, surface of single-walled CNTs was modified with biantennary carbohydrates in order to increase biocompatibility and solubility of CNTs. The cavities of CNTs were filled with Na^125^I and the developed CNT-based radiocarriers were intravenously administered in mice and tracked with CT. It has been demonstrated that the tunability of CNT surface modification offers versatility of organ specific delivery of radionuclides [222]. Ruggiero et al. developed CNT-based carriers to deliver ^225^Ac into the tumor angiogenic vessels, which express the monomeric vascular endothelial-cadherin. Antibody and chelating molecules coating significantly decreased toxicity of the delivery platform as shown in the murine model [223].

Likewise CNTs, pristine graphene and graphene oxide (GO) can induce certain toxicity, however, surface coating of graphene and GO by biocompatible polymers reduces their toxic effects [224, 225]. Therefore, graphene-based materials have been also successfully employed to deliver radionuclides in vitro and in vivo. Indeed, in the recent study Chen and co-workers developed graphene-based radionuclide carriers (^131^I) coated with PEG to reduce their toxicity. Synthesized delivery platforms demonstrated increased efficiency against cancer cells compared with the free ^131^I isotopes [226]. Surface functionalization can not only reduce toxic effects of graphene materials but also increase affinity of radionuclide labeling. Indeed, non-covalently PEGylated GO nanoplatelets demonstrated increased labeling with ^166^Ho [227]. In another work, Zhao et al. proved that polydopamine surface functionalization of GO resulted in enhanced adsorption of U(VI) [228].

Other carbon-based material, nanodiamonds, is generally considered to be non-toxic and biocompatible in various cell types [229]. Therefore, this material can potentially be a good candidate to deliver radiotherapeutics into cells and tissues. Rojas et al. labeled nanodiamonds with functionalized amino groups with ^18^F and monitored the biodistribution of developed systems in rats and mice. Authors observed changes in the biodistribution of the developed formulations by the addition of surfactant agents in suspension of nanodiamonds, [230]. In another study, aminated nanodiamonds were modified with ^223^Ra and ^211^Pb radionuclides with the 93% and 94% of labeling efficiency respectively [231].

### Inorganic NPs labeling approaches

While radiolabeling of antibodies and organic nanoparticles is mostly limited to the use of chelating agents, modification of inorganic NPs with radionuclides does not always require them. For the chelator-based labeling the suitability of the chelator agent for the radionuclide is essential. Prior the conjugation with the chelator, the surface of NPs should be modified with addition of carboxyl, thiols and amino groups. After that the further radiolabeling process is relatively straightforward. Since most of the chelators, such as DOTA, NOTA, BMEDA, and so forth are reactive to carboxyl, thiol and amine groups, they can be incubated with the modified NPs until the conjugation is complete. Afterwards, using the same strategy, NPs can be simply shaken with radionuclides in an aqueous solution. The main drawback of the chelator-based functionalization is that there is no universal chelating agent suitable for every isotope, which means a proper chelator should be selected prior labeling to achieve an efficient modification [232]. However, such surface modification changes the pharmacokinetics of NPs, which can reduce the overall effectiveness of radionuclide delivery. Another setback of the chelator-based labeling is the possible detachment of radioisotope, which decreases specificity of delivery and therefore hampers the potential therapeutic and diagnostic effect of a delivery system [233].

**Fig. 10:**
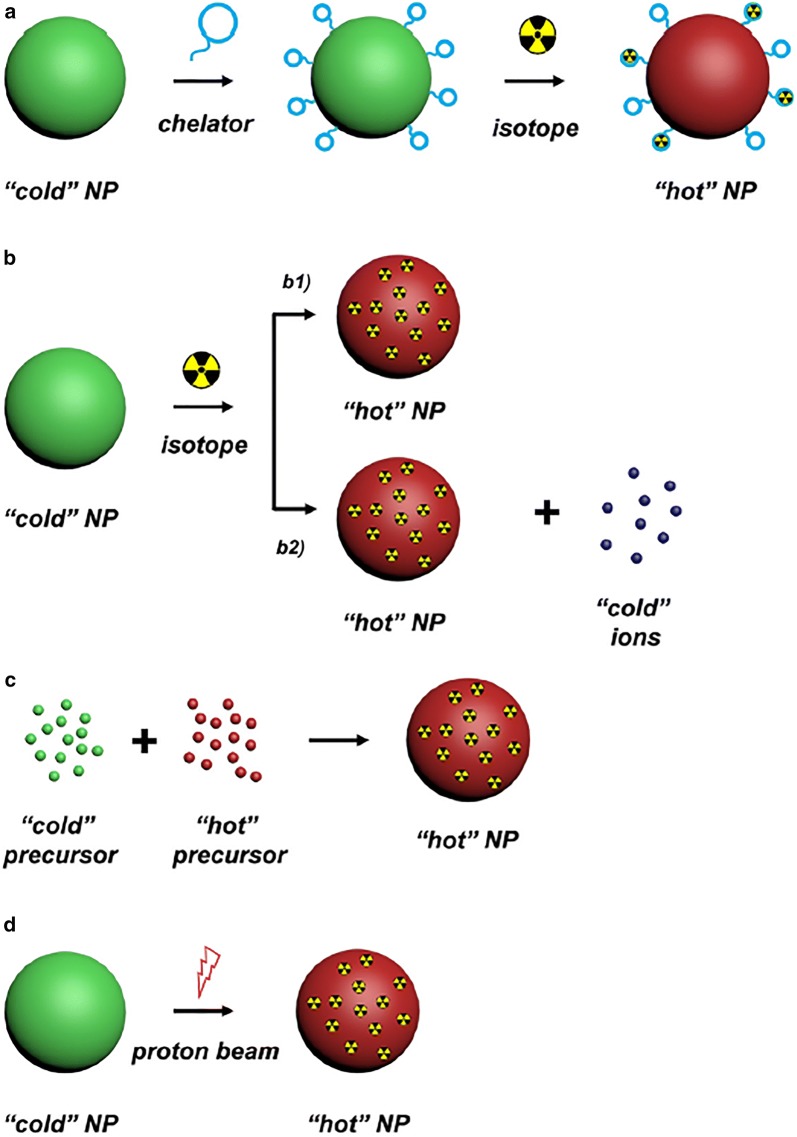
Schematic illustration of five major strategies for radiolabeling inorganic NPs. **a** Chelator-mediated complexation, **b** Specific trapping (b1) and ion-exchange (b2). **c** Hot-plus-cold precursor synthesis, **d** Proton beam activation. NP: nanoparticle (this figure was reproduced from Goel et al. [234] with the required copyright permission)

Therefore, scientific community is also focused on the development of chelator-free labeling approaches. Chelator-free radionuclide labeling is subdivided on “hot-plus-cold” strategy, specific trapping and cation exchange (Fig. 10) [234, 235]. In the “hot-plus-cold” labeling approach radioactive compound is incorporated into the NPs during the synthesis procedure. Such method allows to obtain exceptionally stable labeling with high radiochemical yields. For example, Zhao et al. reported the ^64^Cu-alloyed AuNPs obtained via “hot-plus-cold” synthesis, showed increased radiolabeling stability [236]. However, the main drawback of this technique it that it can be used only for certain combinations of radionuclide and NPs. Additionally, the synthesis should be shorter than the half-life of chosen radionuclide in order to preserve its functionality [237]. The specific trapping method is based on the ability of some metal ions (in case of metallic nuclides) to form stable bonds with oxygen atoms, which are usually expressed on the surface of some inorganic NPs. Like for “hot-plus-cold” approach, specific trapping requires appropriate combinations of radionuclides and NPs. Moreover, for the in vivo applications, the stability of formed bond should be previously checked. Cation exchange labeling technique replaces an ion in a NP by another ion from the solution. This mechanism is driven by the relative thermodynamic stability of the reactants compared to the products. For this the appropriate solvent should be considered. As for other labeling methods, a specific combination of NP-cation pairing is of great importance for the cation exchange labeling.

As an example of chelator-free radionuclide labeling, Chen et al. developed Si-based delivery systems labeled with ^89^Zr using deprotonated silanol groups inside mesopores or on the surface of silica NPs as inherent hard oxygen donors for radiolabeling. The resulted systems demonstrated enhanced stability of ^89^Zr in mice over 3 weeks [238]. Another group showed efficient chelator-free labeling of amorphous Si NPs with clinically relevant radioisotopes (^89^Zr, ^68^Ga, ^111^In, ^90^Y, ^177^Lu, ^64^Cu) by simple adsorption of radionuclides into the NP’s surface. In vivo stability of developed systems was demonstrated and the binding stability was found to be correlated with the hardness of the radioisotope [239]. The same group developed the radiolabeling methods of softer isotopes like ^64^Cu by functionalization of silica NPs with thiol groups. The labeling efficiency was improved from 74.4% for uncoated silica NPs to 94.5% for sulfur coated silica NPs due to the formation of thermodynamically stable bond [240].

#### Outlooks

The inorganic NPs can possess unique physicochemical properties, which can be tuned and optimized by choosing appropriate core material. Therefore, it is possible to find an optimal type of NPs, which will provide required functionalities of radionuclide delivery systems. In other words, apart from radionuclide delivery, NPs with magnetic and plasmonic properties make possible simultaneous visualization of developed radiopharmaceuticals by means of MRI or X-ray imaging. Majority of carbon-based delivery carriers can be visualized with optical fluorescence microscopy, however, to reduce toxic effects of these materials an additional surface modification is required. Silica-based carriers enables high loading capacity due to its porosity and wide functionalization possibilities, nevertheless, extra labeling of these carriers with, for example, fluorescence moieties make it possible optical biodistribution monitoring of such formulations.

The ability to perform chelator-free radiolabeling of inorganic NPs allows to achieve more stable incorporation of radionuclides, which reduces off target effects and overall increases amount of delivered cargo.

However, the toxicity of inorganic NPs is still a topic of research. Therefore, despite variety of inorganic NPs and possibilities of their functionalization, nowadays, they are mostly employed in pre-clinical studies with only a few types of inorganic NPs are clinically approved (ultrasmall Si NPs and Fe_3_O_4_ NPs as contrast agent).

Additionally, inorganic NPs much less effective for targeting of single tumor cells or tumors with lack of vasculature comparing to the mAbs and their fragments. This means that NPs cannot be employed for treatment of metastatic tumors, due to overall inefficiency [237].

The first steps toward clinical applications of radiolabeled inorganic NPs have been made in order to understand the biodistribution of these carriers inside the human body. There are a number FDA-approved inorganic platforms labeled with radionuclides, which were used for medical imaging. Despite the abovementioned ultrasmall Si NPs, ^99m^Tc-labeled sulfur and stannous fluoride colloids were reported to be used as imaging agents in humans [241]. Therefore, we believe that the clinical employment of the inorganic NPs in the radiomedicine will seriously increase, due to the multimodal imaging, which these particles can offer, as well as fine tuning of their physicochemical properties, which helps to predict the biological cell response and the possible clinical outcomes. The recent studies on radionuclide delivery using inorganic NPs were presented in Table 3.

**Table 3:** Recently studied radionuclide delivery systems based on inorganic NPs

Radionuclide	Delivery system	Labeling	Application	Comments	Refs.
^225^Ac	Gold coated lanthanide phosphate NPs	‘Hot’ + ‘cold’ precursor	Radiotherapy	McLaughlin et al. developed multilayered NPs that can deliver multiple a radiations and contain the decay daughters of ^225^Ac while targeting biologically relevant receptors in a female BALB/c mouse model	[200]
^198^Au	Au NPs	‘Hot’ + ‘cold’ precursor	Radiotherapy	Katti et al. developed a novel approach to treat prostate cancer with ^198^Au NPs, which were modified with glucoside molecules mangiferin. In vivo experiments on severe combined immunodeficient (SCID) mice revealed over 85% reduction of bearing prostate tumor volume as compared to the control groups	[201]
^166^Ho	Garnet magnetic NPs	Neutron activation	Radiotherapy	Munaweera et al. developed magnetic NPs, which were modified with ^165^Ho/cytostatic drug and navigated into tumor area using external magnet. In vitro and in vivo results suggested that the external magnetic field navigation was achieved, since higher amounts of ^165^Ho were observed in tumor than in liver, which led to the inhibition of tumor growth	[206]
^89^Zr	Ultrasmall silica NPs	Chelation (DFO)	PET and optic imaging	Chen et al. described synthesis of a PEGylated ultrasmall silica particles labeled with Cy5 dye. Reported NPs were modified with chelators and anti-HER2 scFv fragments. This allowed to achieve more efficient tumor-targeting with bulk renal clearance, which surpasses already existing characteristics of scFv molecules and scFv-conjugated NPs larger than 10 nm. The experiments in vivo revealed a favorable pharmacokinetics and clearance with decreased hepatic and reticuloendothelial system uptake	[212]
^99m^Tc	Mesoporous silica NPs	Reduction of Tc(VII) in TcO_4_^−^ using SnCl_2_	SPECT	Pascual et al. improved targeting properties of developed Si-based systems by functionalization of NPs with Mucin 1 aptamer that is specific to breast cancer. Pronounced targeted delivery of ^99m^Tc into tumor of tumor-bearing Balb/c mice was shown and monitored with SPECT and bioluminescence options	[214]
^89^Zr	Mesoporous silica NPs	Chelation with the deprotonated silanol groups	Photodynamic therapy	Kamkaew et al. reported mesoporous silica NPs loaded with ^89^Zr and photosensitizer Chlorin e6, which can be activated by Cherenkov radiation from ^89^Zr. Therefore, the developed radionuclide delivery systems overcame the limitations of conventional photodynamic therapy, which requires light source to activate photosensitizer	[216]
^225^Ac	CNT	Chelation (DOTA)	Radiotherapy	Ruggiero et al. developed CNT-based carriers to deliver ^225^Ac into the tumor angiogenic vessels, which express the monomeric vascular endothelial-cadherin. The developed carrier system was able to achieve 100-fold amplified cargo delivery (relative to the gold standard for targeted therapy—IgG)	[223]
^131^I	Reduced graphene oxide	Standard chloramine-T oxidation method	Photothermal and radiotherapy	Chen et al. developed radio-labeled graphene-derivative, which offers the ability of in vivo tumor imaging, and is able to deliver both photothermal and radiotherapy at the same time in order to achieve synergistic therapeutic effect using a single nanoscale theranostic agent	[226]

### Microspheres

Another radionuclide delivery systems that have been successfully employed in pre-clinical and clinical studies are microspheres with size distribution in the range of 20–60 µm. Microspheres can be functionalized with active ligands to impart them properties of selectivity. Nowadays, there are three types of radioactive microspheres approved for clinical use—TheraSpheres (BTG, London, UK), SIR-Spheres (Sirtex Medical Limited, North Sydney NSW, Australia) and QuiremSpheres (Quirem Medical BV, Deventer, the Netherlands) [242].

These microspheres are usually used in radioembolization—a procedure, where the microspheres are injected via a catheter into the hepatic arteries. The efficiency of such microspheres is ensured by the fact that healthy hepatic tissue receives its supply mostly from the portal vasculature, while the malignant hepatic tumors are fed by arterial blood. Thus, administration of large microspheres through the hepatic artery leads to the clogging of small tumor capillaries that have a diameter of approximately 8–10 µm. As a result of higher concentration of blood vessels in tumor nodules compared to the normal tissue, the major amount of spheres becomes lodged and emit radiation in the tumor site, leaving the healthy parenchyma relatively unaffected [242–245].

All three clinically approved types of microspheres share a common mechanism of tumor targeting, each of them differs in the means of fabrication, radioactive labeling and choice of materials. These materials possess different physical traits and are applied in slightly different medical conditions.

TheraSpheres are insoluble glass beads with ^90^Y as an integral component and with diameter ranging between 20 and 30 µm. The synthesis of glass microspheres can be divided into two main stages: the particle production and radionuclide activation. The beads are obtained by melting at a high temperature a mixture of ^89^Y oxide (^89^Y_2_O_3_), aluminum oxide (Al_2_O_3_) and silicon dioxide (SiO_2_). The resulting glass is crushed, processed with a flame sprayer to even out the particles and passed through sieves to pick out particles with required distribution. The second stage consists of converting the non-radioactive ^89^Y to ^90^Y by neutron bombardment in a nuclear reactor. The production results in the formation of particles with ^90^Y as an integral component [246]. Such method allows incorporating relatively high amounts of yttrium. The combination of high activity and the particular size distribution allows to minimize the embolic effect, reducing the risk of reflux or stasis/retrograde flow [245, 247, 248].

The SIR-Spheres are microspheres based on biocompatible resin with ^90^Y impregnated on the particle surface. Each bead has a diameter ranging from 20 to 60 µm, and specific activity of 50 Bq at calibration. The activity of each particle, which is relatively small compared to the TheraSphere, is explained by the means of radionuclide incorporation: the ^90^Y is bound exclusively to the resin surface in the form of ^90^Y sulphate.

QuiremSpheres are poly-l-lactic acid based microspheres, containing ^165^Ho. The size of the particles varies from 15 to 60 µm and the use of poly-l-lactic acid allows to achieve the particle density of 1.4 g/cm^3^, which is closer to the density of blood (1.06 g/cm^3^). The ^165^Ho is activated to ^165^Ho with neutron activation in a nuclear reactor. Due to the short half-life period of ^165^Ho, each patient dose of QuiremSpheres needs to be prepared separately, thus, a specific activity can differ in each dose and depends on the needs of a patient [243, 249].

Another difference between yttrium-based microspheres and QuiremSpheres is the biodegradability of the latter. While TheraSpheres and SIR-Spheres remain in the patient liver as a permanent implant, the QuiremSpheres disintegrate leaving only insoluble holmium lactate [250]. Such degradation of microspheres positively influences the outcome of the treatment and enhances the efficiency of consecutive procedures of radioembolization whether they would be required in the future.

### Choice of radionuclide

The choice of ^90^Y as a radionuclide for TheraSpheres and SIR-Spheres was based on its pure β-emitting properties with tissue penetration range of ^90^Y β-radiation of 2.5 mm (max. 11 mm), which in combination with targeting mechanism majorly restrict irradiation mostly to malignant tissues. In addition to that, ^90^Y has a physical half-life suitable for the radiotherapy (64.1 h) and 94% of radiation is delivered within 11 days after the drug admission [251].

**Fig. 11:**
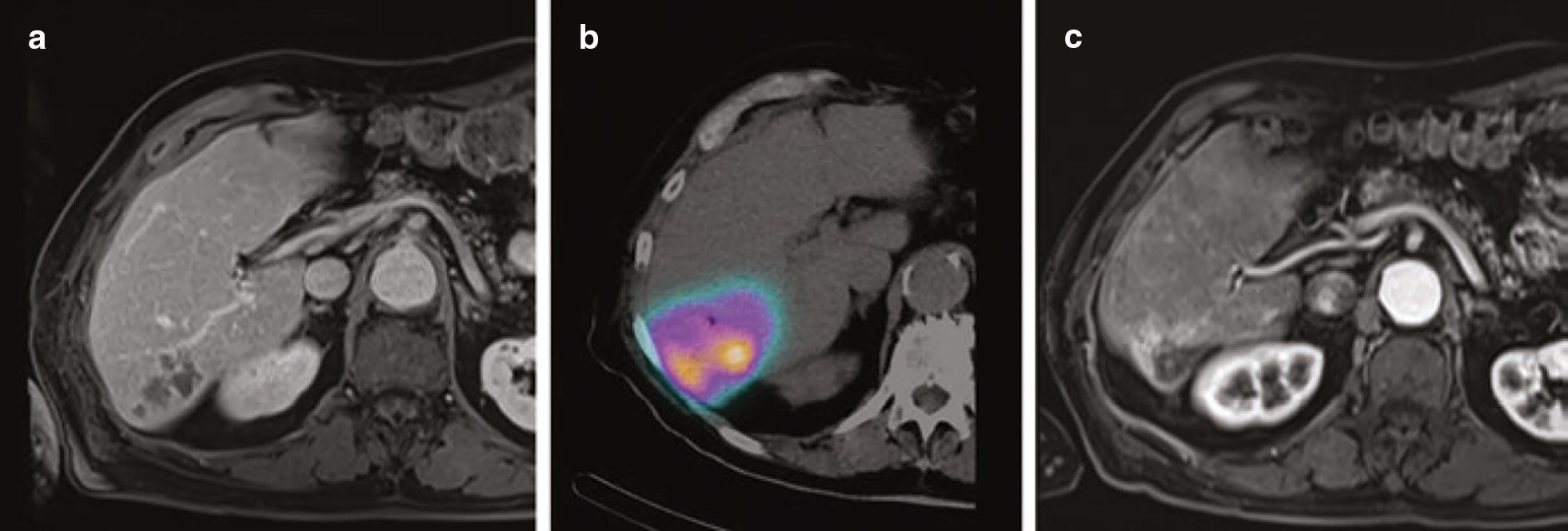
Superselective segmental radioembolization. **a** CT scan before treatment showing multinodular tumor. **b** PET scan showing intense radiation after administration of ^90^Y resin microspheres. **c** Significant atrophy of liver segment and lack of tumor activity 1 year after treatment (this figure is reproduced from Sangro et al. [253] with required copyright permission)

The post-procedural visualization of microsphere distribution is commonly performed by analyzing the bremsstrahlung radiation produced by the decelerating β^−^, but such imaging lacks required precision in dose calculation and evaluation. However, the ^90^Y decay is accompanied by positron emission, which is although relatively weak (0.003%), but still can be detected with highly sensitive enough tomographs, thus, making PET possible with specific equipment (Fig. 11) [252].

Although ^90^Y-based microspheres have proved to be overall effective for hepatic tumor treatment, two major drawbacks lead to the development of ^166^Ho microspheres. Firstly, in order to achieve a therapeutic amount of ^90^Y, long neutron activation times (more than 2 weeks) are required. Secondly, it was proved difficult to track the biodistribution of yttrium microspheres, due to the fact ^90^Y being an almost pure β^−^ emitter [254].

^166^Ho emits low energy γ rays, which accompany electron radiation, when it reaches its steady state as daughter product ^166^Er. This makes possible the visualization of microsphere distribution by SPECT/CT. In addition, both holmium and its decay product erbium are lanthanides, which makes possible the visualization of microsphere by the means MRI of the paramagnetic metal [255].

#### Outlooks

Microspheres have already been successfully implemented in clinical practice long time ago and the recent developments of QuiremSpheres shows that the scientific community is currently developing new approaches in microspheres employment as radionuclide carriers. However, it is important to mention that implementation of microspheres is strictly limited to the radioembolization procedure, which heavily relies on the peculiar blood supply of liver cancer, and, therefore, cannot be used for the treatment of other oncological diseases. Further improvements of the microspheres can be focused around the enhancement of microsphere visualization, which would allow more precise estimation of the delivered radiation dose. In addition, modifications of sphere composition can be made in order to ensure the microsphere degradation and subsequent excretion.

## Conclusion and future prospects

The radionuclides and their capacity to emit ionizing radiation have been employed in clinical practice not only for diagnostic, but also for therapy, with relevant contribution in the several fields of medicine. Given the nature of radioisotopes, as well as the desired goal (e.g. biochemical pathway investigation of radionuclide, bioimaging and anomaly detection, curative or palliative treatment or for theranostic purpose), an appropriate carrier platform should be attentively evaluated and suggested. Due to the advances in chemistry and cellular biology, many radionuclide delivery systems have been developed and demonstrated its efficiency compared to individual alternatives in vitro and in vivo. In this review article, we consider the most trendy radionuclide delivery systems, which can be categorized into four groups: (i) antibodies and their fragments, (ii) organic and (iii) inorganic nanoparticles, and (iv) microspheres. The translation of most of them into clinics has not progressed as rapidly as expected, since each of these platforms possess advantages and disadvantages.

Antibody-based delivery systems are widely employed in therapy and diagnostics due to their distinct pharmacokinetics: highly specific binding to the antigens, easy clearance because of relatively small size. The use of intact antibodies is restricted by poor penetration into solid tumors, whereas the immune system undergoes Fc-mediated bystander activation. However, this limitation can be overcome with antibody fragments. Nevertheless, due to the ultrasmall size of antibody fragments it is not always possible to perform effective labeling with radiometals, since pharmacokinetics of antibodies are affected by incorporating of chelators.

The use of NPs as radionuclide delivery platforms is rapidly developing field due to their unique physicochemical and biological characteristics. To date, many efforts are focused on the translation of developed radiopharmaceuticals based on NPs into clinical practice. However, their fully introduction in clinics is postponed due to technical and biological limitations. There is need for the development of more facile procedures for the surface modification and radiolabeling of NPs. At the same time, biological barriers can induce the different response to the same nanomaterials from patient to patient. The additional stabilization of NPs are highly required, since after the first contact of NPs with the biological fluids organic compounds tend to bind to the NPs surface forming so-called “corona” around the particles. This corona can induce NPs aggregation and significantly change pharmacokinetic profile of NP-based radiopharmaceuticals.

Compared to the inorganic NPs, there are some example of successful clinical trials of organic NPs, especially liposomes ([^64^Cu]MM-302 and ^188^Re-BMEDA liposomes). The clinical implementation of inorganic NPs is limited by concerns over their toxicity, non-optimal utilization and in vivo behavior. As an advantage, inorganic NPs provide additional possibilities of multimodal imaging, for examples, magnetic NPs as contrast agent for MRI, Au NPs as contrast agent for X-ray imaging, C-based NPs as contrast agent for fluorescence imaging. Inorganic and organic NPs enable high radionuclides payloads, flexibility of radiolabeling options and controlled physicochemical parameters (size, shape, chemical composition, stiffness), what results in increased amount of delivered radionuclides in the site of interest and improved tissue penetration and, thus, the efficiency of radiotherapy.

It seems that the scientific community is now at the stage of the developing the advanced hybrid radionuclide carriers with complex architecture taking advantages from each individual delivery system. The integration of various organic and inorganic NPs (e.g. liposomes, micelles, metal NPs, polymer conjugates etc.) in single smart platform allows to achieve multifunctionality and enable multimodal imaging. It also leads to further increase the loading capacity, enhance the biomembrane-crossing rate, and the tissue-penetration efficiency. At the same time, the functionalization of this hybrid platform with targeting moieties may enable the increased targeting delivery of radionuclides.

## Data Availability

Not applicable.

## Abbreviations

NIS: expressing sodium/iodide symporter
DNA: deoxyribonucleic acid
LET: linear energy transfer
SPECT: single-photon emission computed tomography
PET: positron emission tomography
PET/CT: positron emission tomography/computed tomography
Fab: antigen-binding fragment
Fc: fragment crystallizable
mAbs: monoclonal antibodies
RIT: radioimmunotherapy
AUC: area under curve
FcRn: neonatal Fc receptor
scFv: single-chain variable fragments
sdAbs: single domain antibodies
WT: wild type
DM: double mutant
ID/g: injected dose per gram
V_H_: heavy chain variable
V_L_: light chain variable
CEA: carcinoembryonic antigen
PSCA: prostate stem-cell antigen
PSMA: prostate-specific membrane antigen
FDG: fluorodeoxyglucose
TAG-72: tumor-associated glycoprotein 72
IgG: immunoglobulin G
CDR3: complementarity-determining region 3
EGFR: epidermal growth factor receptor
HER2: human epidermal growth factor receptor 2
DTPA: diethylenetriaminepentaacetic acid
^131^I-SGMIB: *N*-succinimidyl 3-guanidinomethyl-5-[^131^I]iodobenzoate
bsAbs: bispecific antibodies
NHS: *N*-hydroxysuccinimide esters
SCN: isothiocyanates
TROP2: trophoblast antigen 2
HSG: histaminesuccinyl-glycine
DFO: desferrioxamine
CPTA: 4-(1,4,8,11-tetraazacyclotetradec-1-yl)-methyl benzole acid tetrahydrochloride
FDA: Food and Drug Administration
NPs: nanoparticles
PEG: poly(ethylene glycol)
NODAGA: 2-(4,7-bis(carboxymethyl)-1,4,7-triazonan-1-yl)pentanedioic acid
DOTA: 1,4,7,10-tetraazacyclododecane-1,4,7,10-tetraacetic acid
NOTA: 1,4,7-triazacyclononane-1,4,7-triacetic acid
BMEDA: *N*,*N*-bis(2-mercaptoethyl)-*N*’,*N*’-diethylethylenediamine
HMPAO: hexamethylpropyleneamine oxime
MRI: magnetic resonance imaging
EPR: enhanced permeation and retention
HSA: human serum albumin
BSA: bovine serum albumin
SLN: sentinel lymph node
DTPA: diethylenetriaminepentaacetic acid
HSA: human serum albumin
PAMAM: polyamidoamine
PPI: polypropyleneimine
PLL: poly-l-lysine
MMP-2: matrix metalloproteinase-2
CTX: chlorotoxin
HPAO: 3-(4′-hydroxyphenyl)propionic acid-OSu
H_4_do3a-py^NO‑C^: 10-[(4-carboxy-1-oxidopyridin-2-yl)methyl]-1,4,7,10-tetraazacyclododecane-1,4,7-triacetic acid
CT: computer tomography
NIR: near infrared
DTPA-PE: DTPA-phosphatidylethanolamine
DTPA-SA: DTPA-stearylamine
CB-TE2A: 2,2′‐(1,4,8,11‐tetraazabicyclo(6.6.2)hexadecane‐4,11‐diyl)diacetic acid
pHEMA: poly(2-hydroxyethyl methacrylate)
PCMA: poly(4-methylcoumarin methacrylate)
DMF: dimethylformamide
PBLG: poly(γ-benzyl l-glutamate)
PLA: polylactic acid
PGA: polyglycolic acid
PLGA: poly(lactic-co-glycolic acid)
PEO: polyethylene oxide
Au NPs: gold nanoparticles
SCID: severe combined immunodeficient
SPIONs: superparamagnetic iron oxide nanoparticles
ICG: indocyanine green
CNTs: carbon nanotubes
Cy5.5: cyanine5.5
CDs: carbon dots
GO: graphene oxide
