# Correction to: Current outlook on radionuclide delivery systems: from design consideration to translation into clinics

**DOI:** 10.1186/s12951-019-0558-z

**Published:** 2020-01-02

**Authors:** Oleksii O. Peltek, Albert R. Muslimov, Mikhail V. Zyuzin, Alexander S. Timin

**Affiliations:** 1 Russian Research Center of Radiology and Surgical Technologies (RRCRST) of Ministry of Public Health, Leningradskaya Street 70 Pesochny, Saint-Petersburg, 197758 Russian Federation; 2 Faculty of Physics and Engineering, ITMO University, St. Petersburg, 197101 Russia; 3 Research School of Chemical and Biomedical Engineering, National Research Tomsk Polytechnic University, Lenin Avenue 30, Tomsk, 634050 Russia

## Correction to: J Nanobiotechnol (2019) 17:90 10.1186/s12951-019-0524-9

After publication of this article [1], an error was found in the description of the holmium isotopes. ^165^Ho is a stable isotope a fraction of which is activated to ^166^Ho by neutron activation in a nuclear reactor [2]. In one paragraph of the published article, describing holmium containing QuiremSpheres, ^165^Ho should be replaced with ^166^Ho. The correct description is given below:

“QuiremSpheres are poly-l-lactic acid based microspheres, containing ^166^Ho. The size of the particles varies from 15 to 60 μm and the use of poly-l-lactic acid allows to achieve the particle density of 1.4 g/cm^3^, which is closer to the density of blood (1.06 g/cm^3^). The ^165^Ho is activated to ^166^Ho with neutron activation in a nuclear reactor. Due to the short half-life period of ^166^Ho, each patient dose of QuiremSpheres needs to be prepared separately, thus, a specific activity can differ in each dose and depends on the needs of a patient.”
